# Young adult carers' identification, characteristics, and support: A systematic review

**DOI:** 10.3389/fpsyg.2022.990257

**Published:** 2022-10-24

**Authors:** Basilie Chevrier, Kristopher Lamore, Aurélie Untas, Géraldine Dorard

**Affiliations:** ^1^Aix-Marseille Université, PSYCLÉ, Aix-en-Provence, France; ^2^Université de Lille, CNRS, UMR 9193 - SCALab - Sciences Cognitives et Affectives, Lille, France; ^3^Université Paris Cité, Laboratoire de Psychopathologie et Processus de Santé, Boulogne-Billancourt, France

**Keywords:** emerging adulthood, informal carers, prevalence, physical and psychological health, support service access

## Abstract

Young Adult Carers (YAC) are informal carers aged 18–25 years. This is an unrecognized population. The present systematic review aims to respond to: (1) how YAC are identified in research; (2) the prevalence of YAC; (3) the characteristics of YAC; and (4) how to support YAC. Five electronic databases (Google Scholar, PsycArticle, PsycInfo, Psychology and Behavioral Sciences Collection, and PubMed) were searched for studies, scientific articles, and gray literature on YAC published prior to January 18, 2021. The quality of included studies was assessed using the Crow Critical Appraisal Tool, and a narrative method was used to underline major findings. Twenty-three studies were included and revealed that there were several ways to identify YAC, resulting in heterogeneous prevalence. Nine themes were highlighted for YAC characteristics (way into caring; care receiver; caring responsibilities; amount of caring; self-identification as a carer; living arrangement; physical, psychological, and adaptative outcomes; interpersonal relationships; education and employment); and three for YAC support (needs, available support services, and recommendations). The findings showed the diversity of YAC experiences. Although YAC expressed several needs, there are few or no support services devoted to them. More research is needed to improve political awareness.

**Systematic review registration:**

https://www.crd.york.ac.uk/prospero/display_record.php?ID=CRD42021231882, identifier: CRD42021231882.

## Introduction

When a person provides regular, non-professional assistance with daily activities or emotional support to a relative or a family member who has an illness, disability, or loss of autonomy due to age, he/she is called a carer (Blanc, 2010). Carers are often adults but can also be children, adolescents, or young adults. Young adult carers (YAC) have been defined as “people age 18–24 who provide or intend to provide care, assistance or support to another family member on an unpaid basis. The person receiving care is often a parent but can be a sibling, grandparent, partner, own child or other relative who is disabled, has some chronic illness, mental health problem or other condition (including substance misuse) connected with a need for care, support or supervision” (Becker and Becker, 2008a, p. 6). This definition can be extended to young people up to age 25, in light of emerging adulthood theory (e.g., Levine et al., 2005; Lewis, 2017). According to emerging adulthood theory, young people between 18 and 25 years old are in a distinct stage of life; they are neither adolescent nor adult (Arnett, 2000). Emerging adulthood can be seen as an “age of possibilities” defined by progressive autonomy, relative independence from social roles, self-exploration, and new experimentation (Arnett, 2004).

Levine et al. (2005) identified YAC as an unstudied population. Ten years later, Day (2015a) highlighted the need to specifically target YAC as a distinct cohort. More recently, Kent (2020) underlined that it is time to recognize and support YAC. The present systematic review aims to respond to (1) how YAC are identified in research; (2) the prevalence of YAC; (3) the characteristics of YAC; and (4) how to support YAC.

## Methods

This review was guided by the Preferred Reporting Items for Systematic Review and Meta-Analysis Statement (PRISMA; Page et al., 2021). See Supplementary material 1 for the PRISMA checklist. The study has been registered on PROSPERO under number CRD42021231882.

### Search strategy and eligibility

We queried the following electronic databases: Google Scholar, PsycArticle, PsycInfo, Psychology and Behavioral Sciences Collection, and PubMed. This research included articles written in English and in French that pertained to YAC ages 18–25 and were published up to January 18, 2021. Considering the research questions, a list of search terms was developed as follows: “young adult carers,” “young adult caregivers,” “emerging adult carers,” “emerging adult caregivers,” “student carers,” and “student caregivers.” Records were identified through database searching on January 19, 2021, for Google Scholar and January 20, 2021, for PsycArticle, PsycInfo, Psychology and Behavioral Sciences Collection, and PubMed.

Both full scientific articles and gray literature were considered. Full scientific articles could be quantitative, qualitative or mixed-method study as well as literature review or meta-analysis. Gray literature could be public reports, master or dissertation thesis or scientific communications. To be included, an article had to be exclusively about young adults aged 18–25 who currently provide informal (unpaid) care, assistance, or support to a family member or relative. As facing with the illness/disability of a relative did not necessarily lead to endorse a caring role (Becker, 2007; Chevrier et al., 2022c), we only consider studies which defined YAC as informal carers. Studies with samples integrating YAC (18–25 years) and other caregivers in separate groups were also included. Studies were excluded when they did not consider YAC (18–25 years) as a distinct group. All variables related to YAC were examined. The selection process was as follows: first, we read through all the titles and abstracts. Second, when the documents seemed to match but the information on the age population was missing, we read the methodology section. Finally, a list of eligible studies was established after full-text reading. BC performed all database searching and selection under the supervision of GD and AU.

### Data extraction and quality assessment

A table was created with the following information on the selected studies: authors, type of document, study design, years, countries, population age, population sociodemographic information, care receivers' types of illness, caring activities, YAC identification procedures, measures, strengths, limitations, and key findings. These outcome domains were chosen in regard to our research questions. This data collection process was conducted by BC under the supervision of GD and AU. The findings were then explored using a narrative method (Baumeister and Leary, 1997) that allows synthesis of information by gathering study findings. A preliminary list of themes was generated by grouping similar findings, and this list was then discussed and organized into major themes related to our research questions. In addition, the Crowe Critical Appraisal Tool (CCAT) was used to test the quality of each study and assess its risk of bias (Crowe, 2013). This procedure was used to assign each study quality rating. Each study was scrutinized on the quality of its preliminaries (title, abstract), introduction, design, sampling, data collection, ethical matters, results, and discussion. This procedure was followed by BC and KL independently under the supervision of GD and AU.

## Results

### Study selection, characteristics, and quality

**Figure 1:**
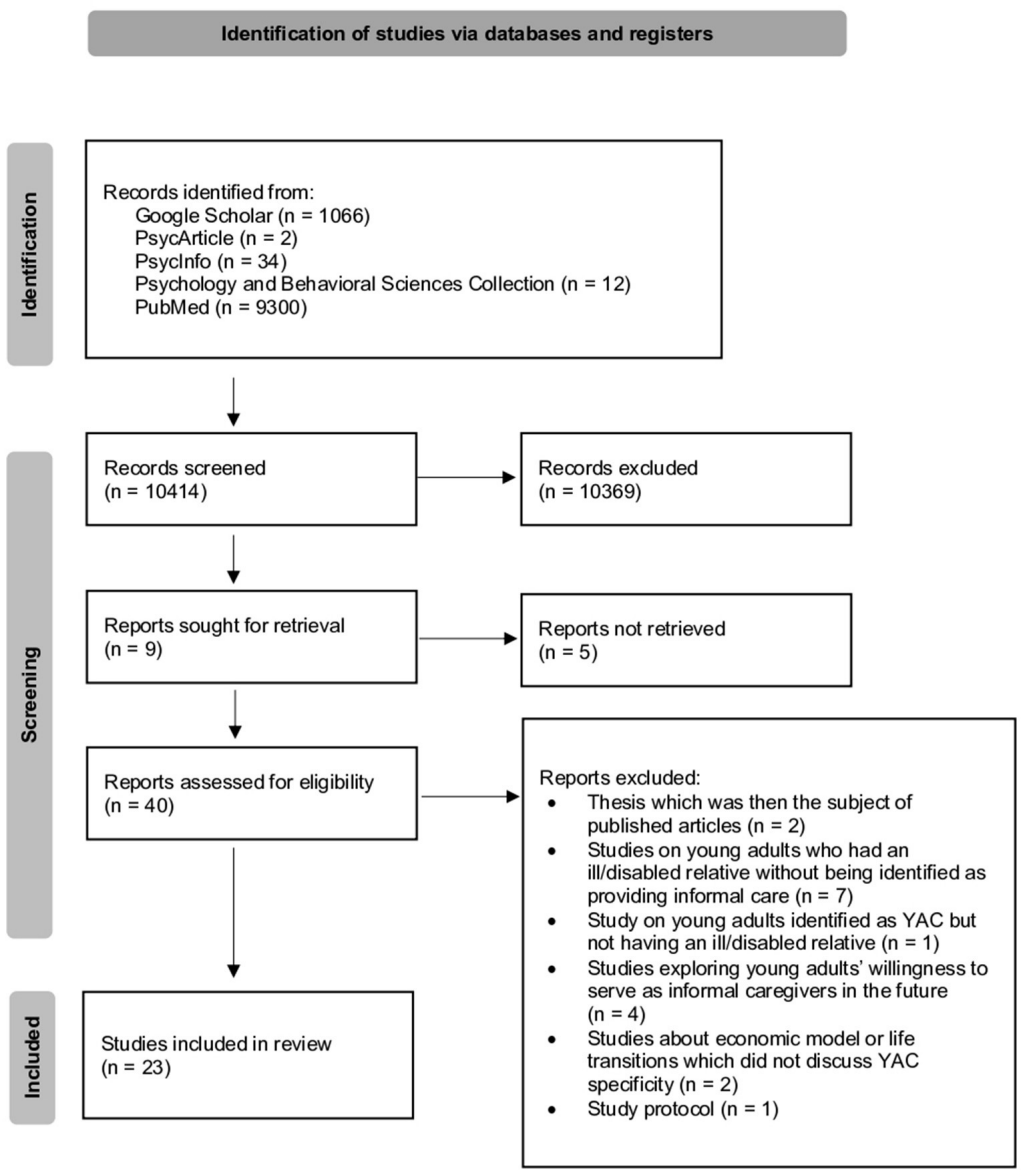
PRISMA flow diagram.

The database search and identification procedure led to 10,414 studies. After screening, 40 studies were examined in full. During full-text screening, 17 studies were excluded for the following reasons: thesis that became the subject of published articles (*n* = 2; Abraham, 2010; Joshi, 2014); studies on young adults who had an ill/disabled relative but were not identified as providing informal care (*n* = 7; Abraham and Stein, 2013, 2015; Mechling, 2015; Petrowski, 2015; Hinojosa et al., 2018; Nuttall, 2018); a study on young adults identified as YAC but without an ill/disabled relative (van der Werf et al., 2020b); studies exploring young adults' willingness to serve as informal caregivers in the future (*n* = 4; Alva, 2013; Joshi et al., 2015; Trujillo et al., 2016; van der Werf et al., 2020a); studies about economic or life transitions that did not discuss YAC specifically (*n* = 2; González-Arnal and Kilkey, 2009; Day, 2015b); and a study protocol (Leu et al., 2018). As a result, 23 studies were included in this review (Levine et al., 2005; Mancini et al., 2006; Becker and Becker, 2008a,b; Cass et al., 2011; Hamilton and Adamson, 2013; Struckmeyer, 2013; Day, 2015a, 2019; Greene et al., 2017; Lewis, 2017; Thompson et al., 2017; Boumans and Dorant, 2018; Jones, 2018; Kettell, 2018; Leu et al., 2018; Canell and Caskie, 2019, 2020; Canell et al., 2020; Grenard et al., 2020; Haugland et al., 2020; Joseph et al., 2020; Kent, 2020). See Figure 1 for the PRISMA flow diagram and Supplementary material 2 for the list of studies included in the review.

The key characteristics of all studies included in this review are presented in Table 1 and in Supplementary material 3. Of the 23 studies included, there were 12 articles and 11 documents from gray literature: three masters theses or dissertations, two letters, three poster presentations, and three national reports. The data came from the United States (*n* = 9), the United Kingdom (*n* = 5), Australia (*n* = 4), the Netherlands (*n* = 1), Norway (*n* = 1), and Switzerland (*n* = 1). Only one study used data from both the United States and the United Kingdom but did not statistically compare them (Lewis, 2017). In their study design, the documents presented a wide range of methods, with qualitative studies (*n* = 8), quantitative studies (*n* = 7), mixed-method studies (*n* = 4), and literature reviews (*n* = 4). However, they all used a cross-sectional design. The qualitative design included semi-structured interviews, in-depth interviews, focus groups, and open-ended questions, whereas the quantitative design referred to self-reported questionnaires. Data were collected among YAC (*n* = 6), YAC students (*n* = 6), students (*n* = 3), youth (*n* = 2), and adult carers (*n* = 1); one study did not mention if the sample of YAC were students or not (Leu et al., 2018). Fifteen studies presented a sample that was more than 60% female, and three studies only concerned female YAC (Mancini et al., 2006; Jones, 2018; Kettell, 2018). Three studies involved YAC and another caring group: young carers (YC) as informal caregivers under 18 years old (Becker and Becker, 2008a; Cass et al., 2011; Leu et al., 2018). Sample size varied from 3 to 40,205 participants, and the sample size was missing in one study (Canell and Caskie, 2020). Six studies had fewer than 15 participants, four had 16–99, five had 100–354, and three had more than 355. The included studies presented a wide range of quality, from 30 to 93% (see Table 1). Half of the studies had a score higher than 60%.

**Table 1:** Included study type, country, sample, identification procedure, and CCAT scores.

**Author(s)**	**Type / design**	**Country**	**Sample/specialty**	**Identification procedure**	**CCAT**
Becker and Becker (2008a)	Report / 3	UK	Census: *NA* Survey: 359 YC and 95 YAC In-depth interviews: 25 YAC (72% females)	Census: *NA* Survey and in-depth interviews: already identified as YAC	53
Becker and Becker (2008b)	Report / 4	UK	Census: *NA*	Census: being between 18 and 24 years and “looking after or giving any help or support to family members, friends, neighbors or others because of long-term physical or mental ill-health or disability or problems related to old age”	68
Boumans and Dorant (2018)	Article / 1	Netherlands	297 students in health and science university or in nursing school (74.74% females)	Identifying themselves as carer	80
Canell and Caskie (2019)	Poster / 3	USA	213 YAC (60.6% females)	*NA*	60
Canell and Caskie (2020)	Poster / 1	USA	YAC (60% females)	“Unpaid for caregiving tasks, endorse participating in at least one item on a list of caregiving tasks (activities of daily living/ instrumental activities of daily living) for a care recipient over the age of 65”	58
Canell et al. (2020)	Poster / 3	USA	248 YAC (60% females)	“ Unpaid for caregiving tasks, endorse participating in at least one item on a list of caregiving tasks (activities of daily living/ instrumental activities of daily living) for a care recipient over the age of 65”	53
Cass et al. (2011)	Report / 3	Australia	23 YC (34.78% females) and 13 YAC (38.46% females)	Already identified as YAC	58
Day (2015a)	Article / 4	Australia	*NA*	*NA*	48
Day (2019)	Article / 2	Australia	13 YAC students in education, health, law, business, arts and science university (92.31% females)	*NA*	50
Greene et al. (2017)	Article / 1	USA	353 undergraduate students (80.17% females; 196 non-caregivers, 81 past caregivers, and 76 current/past caregivers)	“Do you currently provide assistance to a person who needs special medical care as a result of an injury, aging, illness, disability, or other health condition?”	78
Grenard et al. (2020)	Article / 1	USA	18,237 emerging adults (45.85% females; 3,087 caregivers, 2,303 expectant caregivers, and 12,216 non-caregivers)	“During the past 30 days, did you provide regular care or assistance to a friend or family member who has a health problem or disability?”	83
Hamilton and Adamson (2013)	Article / 2	Australia	36 youth (47.22% females)	Already identified as YAC	45
Haugland et al. (2020)	Article / 1	Norway	40,205 students (70.2% females)	“Had regular care responsibilities for someone with physical or mental illness, disabilities, or substance misuse (not his/her own child/children)”	93
Jones (2018)	Dissertation / 2	UK	5 YAC students (100% females)	Already identified as YAC	65
Joseph et al. (2020)	Article / 4	*NA*	*NA*	*NA*	48
Kent (2020)	Letter / 4	*NA*	*NA*	*NA*	45
Kettell (2018)	Article / 2	UK	3 YAC undergraduate students (100% females)	*NA*	68
Leu et al. (2018)	Article / 2	Switzerland	15 YC (73.33% females) and 14 YAC (92.86% females)	Having a caring role	78
Levine et al. (2005)	Article / 1	USA	National survey Harvard/UHF/VNS: 1,247 adult carers (25.5% females) National survey NAC/AARP: 1,002 adult carers (49.2% females)	“Provides unpaid or arranges for paid or unpaid help to a relative or friend because they have an illness or disability that leaves them unable to do some things for themselves or because they are getting older”	45
Lewis (2017)	Dissertation / 2	UK-USA	55 YAC (27 UK and 28 USA; 69.09% females)	“Providing unpaid care, assistance, and support to a family member who has a health condition requiring care. Conditions may include physical and learning disabilities, mental illness, chronic health issue, and substance misuse”	78
Mancini et al. (2006)	Letter / 2	USA	3 YAC students (100% females)	*NA*	30
Struckmeyer (2013)	Dissertation / 1	USA	118 YAC undergraduate students (78% females)	Identifying themselves as carer	75
Thompson et al. (2017)	Article / 2	USA	98 YAC students (69.38% female)	“Supporting a parent with a chronic health condition” “Chronic means the condition is enduring, persistent, long-lasting, or recurring. The health condition can be anything-visible or invisible, minor or severe, mental or physical”	63

NA, not available; YAC, young adult carers; YC, Young carers; CCAT, Crowe Critical Appraisal Tool; UK, United Kingdom; USA, United States of America.

Study design: 1, Quantitative research; 2, Qualitative research; 3, Mixed-method; 4, Literature review.

The examination of the included studies showed nine themes about YAC characteristics: (1) ways into caring; (2) care receiver; (3) caring responsibilities; (4) amount of caring; (5) self-identification as a carer; (6) living arrangement; (7) physical, psychological, and adaptative outcomes; (8) interpersonal relationships; and (9) education and employment. Three themes about YAC support were identified: (1) needs for support, (2) available support services, and (3) recommendations for support services.

### How YAC are identified in research

The identification procedure used for each study is reported in Table 1. Three identification procedures were used: already identified as YAC (*n* = 4), self-identification (*n* = 3), and criteria based on broad definitions (*n* = 9). The first procedure recruited participants through national services or associations for young carers. The second procedure involved directly asking participants whether they identified as carers. The third procedure included several criteria based on broad definitions that the participant was directly asked about or, in the case of a census, that other adults within the household were asked about. Among these criteria, there were four major themes: nature of support (e.g., “participating in activities of daily living and/or instrumental activities of daily living,” “care, assistance, support”), frequency (e.g., “regular,” “currently”), care receiver (e.g., “friend,” “parent”), and type of illness or disability (e.g., “chronic health condition,” “injury, aging, illness, disability, or other health conditions”). Only one study gave a definition that included all four themes (Grenard et al., 2020). Two studies were partially based on census information (Becker and Becker, 2008a,b). It is worth noting that four empirical studies did not give information on the identification procedure (Mancini et al., 2006; Kettell, 2018; Canell and Caskie, 2019; Day, 2019).

### The prevalence of YAC

The prevalence of YAC reported in the included studies is quite heterogeneous. This heterogeneity may be explained by an absence of a unique definition and thus identification method (Joseph et al., 2020). Only five included studies had the objective of highlighting the prevalence of YAC among youth, adult carer, or student populations (Levine et al., 2005; Becker and Becker, 2008a,b; Grenard et al., 2020; Haugland et al., 2020). Among youth, YAC make up 5.30% in the United Kingdom (16–24 years; Becker and Becker, 2008a,b) and 18.10% in the United States (18–25 years; Grenard et al., 2020). Among adult carers in the United States (18–over 65 years), 12–18% are YAC (Levine et al., 2005). Finally, 5.50% of students in Norway are YAC (18–25 years; Haugland et al., 2020).

The other included studies that examined student populations led us to estimate YAC prevalence within their subsamples. Boumans and Dorant (2018) found 18.85% of YAC in a subsample of Dutch students, and Greene et al. (2017) found 21.53% in a subsample of American students. In addition, a literature review conducted by Day (2015a) suggested that among Australian young carers, two-thirds are YAC.

### The characteristics of YAC

#### Ways into caring

Several factors have been identified to explain why young adults endorse caring responsibilities. These factors could be: the care receiver's illness or disability, dependency and needs; the family structure (i.e., single or divorced parents); the current relationship status of the young adult (i.e., being single); the good quality of relationship with the care receiver and the family; the absence of other informal carers; the positive ageist attitudes (i.e., stereotyping and/or discrimination against individuals on the basis of their age) when the care receiver is a grandparent; the carer's physical proximity and availability; the family's ethnicity (i.e, immigrant or ethnic minority); poor financial circumstances; a lack of suitable formal care arrangements; and the extent to which the care receiver was receiving or accepting support from others (Becker and Becker, 2008a,b; Cass et al., 2011; Struckmeyer, 2013; Boumans and Dorant, 2018; Canell and Caskie, 2020; Canell et al., 2020; Haugland et al., 2020; Joseph et al., 2020). For some studies, gender and birth order were also involved, as more YAC were female than male and more were the eldest child than the youngest (Becker and Becker, 2008a; Struckmeyer, 2013; Haugland et al., 2020). In other studies, however, there were no gender or rank differences (Boumans and Dorant, 2018). In one study, males outnumbered females (Levine et al., 2005). The number of children in the family did not seem to be a factor (Boumans and Dorant, 2018). Among the American youth, 13.90% expected to become a caregiver within the next 2 years; these were mostly male and Hispanic (Grenard et al., 2020).

Three specific ways into caring were highlighted: suddenly due to a significant change in the family structure, gradually as young adults become older, and as a normal part of family life (Lewis, 2017; Leu et al., 2018). For some YAC, it clearly appeared that they became a carer as there was no other option (Day, 2015a; Canell et al., 2020), whereas for others, it was a personal choice and they continued to do it because they wanted to (Hamilton and Adamson, 2013). However, there was more expectation of young adults to endorse a caring role because they were seen as adults and thus more mature and able to cope with it (Becker and Becker, 2008a,b; Cass et al., 2011). Caring responsibilities could be seen as a normal practice that fulfills YAC memberships in their family (Lewis, 2017). The caring role could then be considered a continuing responsibility rather than a phase in the young adult's life, especially if YAC cared for parents or siblings (Cass et al., 2011). Overall, a diversity of experiences led to becoming a carer, and each experience may have had a distinct impact on caregiving behaviors (Canell et al., 2020).

#### Care receiver

YAC appeared to mostly care for one person, but they may care for two or more (Greene et al., 2017; Boumans and Dorant, 2018). If YAC had younger siblings, they took on responsibilities for them as well (Leu et al., 2018). In that way, YAC could care for multiple family members (Becker and Becker, 2008b; Lewis, 2017). Generally, the care receiver was a parent or a grandparent (Greene et al., 2017; Lewis, 2017; Boumans and Dorant, 2018; Canell and Caskie, 2019, 2020; Canell et al., 2020), mostly a mother or a grandmother (Levine et al., 2005; Becker and Becker, 2008a; Thompson et al., 2017). The care receivers could also be part of the immediate family or a close personal friendship like an aunt or uncle, sibling, partner, own child, best friend, or neighbor (Becker and Becker, 2008a; Cass et al., 2011; Struckmeyer, 2013; Lewis, 2017; Boumans and Dorant, 2018; Canell and Caskie, 2019; Canell et al., 2020). In general, in the United States, YAC cared for a person two generations above them (Levine et al., 2005; Canell and Caskie, 2019, 2020; Canell et al., 2020).

The care receivers could suffer from a chronic disease, a physical disability, a mental disorder, an intellectual disability, or an alcohol and substance problem (Becker and Becker, 2008a; Cass et al., 2011; Hamilton and Adamson, 2013; Lewis, 2017; Boumans and Dorant, 2018; Leu et al., 2018; Haugland et al., 2020). Some of the care receivers had comorbidities (Boumans and Dorant, 2018). Only one study was conducted about a specific illness or disability (i.e., breast cancer; Mancini et al., 2006). Chronic disease could be arthritis, dementia, Alzheimer's disease, Huntington's disease, Parkinson's disease, Lewy body dementia, cancer, multiple sclerosis, diabetes, or hypertension (Mancini et al., 2006; Thompson et al., 2017; Jones, 2018; Kettell, 2018; Canell and Caskie, 2019, 2020; Day, 2019; Canell et al., 2020). Physical disability could be related to a general decline due to age or to paraplegia, muscular dystrophy, cerebral palsy, or limited mobility (Cass et al., 2011; Canell and Caskie, 2019; Day, 2019; Canell et al., 2020). Mental disorder could be depression as well as other mental illnesses like bipolar disorder or autism (Thompson et al., 2017; Jones, 2018; Kettell, 2018; Canell and Caskie, 2019; Day, 2019; Canell et al., 2020). Some studies did not clearly identify the care receiver's illness or disability (Becker and Becker, 2008a; Hamilton and Adamson, 2013; Lewis, 2017; Boumans and Dorant, 2018; Leu et al., 2018; Haugland et al., 2020).

#### Caring responsibilities

YAC were mostly primary caregivers (Canell and Caskie, 2019, 2020; Canell et al., 2020) and could be the only caregiver when the caring role was not shared with other family members (Becker and Becker, 2008a; Boumans and Dorant, 2018). Among the student population, YAC could be a secondary or tertiary caregiver (Struckmeyer, 2013). As soon as caring responsibilities were shared with other family members, the amount of caring became less significant for the YAC (Becker and Becker, 2008a). However, being the primary, secondary, or tertiary caregiver all led to strain or overload (Struckmeyer, 2013).

Care receivers often needed assistance in taking medications (Levine et al., 2005) or in a range of health and/or social needs (Becker and Becker, 2008a). YAC were thus involved in a wide range of tasks and responsibilities, which could be considered as a dichotomy of activities of daily living and instrumental activities of daily living (Struckmeyer, 2013; Greene et al., 2017). Activities of daily living included feeding, bathing, or dressing, whereas instrumental activities referred to emotional support, cleaning, cooking, or dispensing medications (Greene et al., 2017).

YAC provided emotional support as well as household chores, intimate and personal care, nursing duties, childcare tasks, or administrative tasks (Becker and Becker, 2008a; Cass et al., 2011; Day, 2015a; Lewis, 2017; Boumans and Dorant, 2018; Leu et al., 2018). Emotional support and care included attending to the care receiver's emotional and psychological wellbeing (Day, 2015a). This seemed to be a difficult task for some YAC, as it restricted their participation in social life events (Becker and Becker, 2008a). When YAC had endorsed the caring role since childhood or adolescence, they explained that emotional care became more important over the past year as they got older (Cass et al., 2011). For YAC students who left the family household to study, emotional support was still a core issue, as a “caring at a distance” task (Becker and Becker, 2008a). Household chores implied cleaning, cooking, or laundry (Day, 2015a). Intimate and personal care referred to helping the care receiver wash or take a bath (Becker and Becker, 2008a). Compared to older carers, YAC were less likely to do this task (Levine et al., 2005). Nursing duties referred to giving medication, changing dressings, and assisting with mobility (Day, 2015a). Childcare tasks referred to supervising siblings and handling school lunches or transportation (Day, 2015a). Finally, administrative tasks included dealing with financial issues and coordinating with healthcare professionals, social services, and other authorities (Leu et al., 2018).

The activities that were the most frequently undertaken were assistance with walking, shopping, feeding, dressing, emotional care, and cleaning (Levine et al., 2005; Greene et al., 2017). The most common activities of daily living were getting out of bed, dressing and toileting (Levine et al., 2005). YAC were also more involved than non-YAC in organizing help from others, coordinating appointments, and administering medication (Greene et al., 2017), as well as managing finances and arranging services (Levine et al., 2005).

Overall, these tasks and responsibilities could be verbal or nonverbal, instrumental or tangible, and informational behaviors (Thompson et al., 2017). All these caring responsibilities contributed to the YAC burden (Struckmeyer, 2013). Moreover, from a lifespan communication perspective, YAC appeared to provide an “understanding” form of social support. YAC had understanding and knowledge about the care receiver's illness or disability and its consequences, and they communicated their understanding through reciprocating support, sacrificing, being obedient, avoiding sensitive topics, and projecting emotional strength. This “understanding” form of social support reflected the emerging adulthood stage of life through the shift of parent-child relationships from hierarchical to reciprocal. In that sense, YAC caring responsibilities reflected their individual development (Thompson et al., 2017).

The activities for which YAC were responsible depended on the nature of the care receiver's illness or disability, the availability of other family carers, and the structure and dynamics of the family (Cass et al., 2011; Leu et al., 2018). Compared to YC, YAC were more likely to perform additional tasks such as medical appointments, talking with health or other service providers, helping with bills, and providing emotional support (Cass et al., 2011). Moreover, YAC students could take an instrumental parental role for their siblings (Boumans and Dorant, 2018). For YAC students who left the family household for university, caring activities became less physical than before (Becker and Becker, 2008a), although compared to non-carer students, they experienced more household chores (Boumans and Dorant, 2018).

The circumstances that led to becoming a YAC were also related to the level of willingness to provide caregiving. YAC who identified family relationships as a circumstance said they were less willing to provide nursing care in the future than others did. In contrast, those who identified the care receiver's dependency as a circumstance said they were more likely to provide instrumental and emotional care in the future than others did (Canell et al., 2020).

#### Amount of caring

The amount of caring activities could vary from a part-time to a full-time responsibility (Leu et al., 2018) and depended on the condition or emotional state of the care receiver as well as on the structure of the family; YAC in a single-parent family structure spent more time caring than YAC with two parents (Becker and Becker, 2008a). The amount of caring increased over time when the care receiver was a parent whose condition deteriorated. Conversely, when the care receiver was a sibling, the amount of caring could decrease over as the sibling became able to do more by himself/herself (Becker and Becker, 2008a). On average, YAC provided between almost 13 and 20 h per week (Becker and Becker, 2008a,b; Boumans and Dorant, 2018). For a few YAC, caring time could exceed 50 h per week (Becker and Becker, 2008a,b).

For YAC students, during a typical school day, the amount of care could span 3–5 h, whereas during the weekend, it could be more than 8 h a day (Greene et al., 2017). Overall, YAC students spent more hours on care responsibilities during the weekend (Haugland et al., 2020). On both weekdays and weekends, YAC student females spent more time on care responsibilities than males (Haugland et al., 2020). Compared to nonstudents, YAC students did not spend less time on caring activities, meaning that after a day at university, they returned to their caring responsibilities (Becker and Becker, 2008a).

Most YAC assumed their care responsibilities over a long period of time (Becker and Becker, 2008a). A large majority started caring before 16 years old, some considerably earlier, but others began after age 16 (Becker and Becker, 2008a; Greene et al., 2017; Lewis, 2017). YAC could fill a caring role for a few months or for 10 years or more (Levine et al., 2005; Struckmeyer, 2013; Boumans and Dorant, 2018).

#### Self-identification as carer

Identifying and assessing YAC is a major problem (Becker and Becker, 2008b). Most YAC did not appear to consider themselves as YAC until someone, most often a healthcare or social professional, defined them as such (Becker and Becker, 2008a; Lewis, 2017). Prior to identifying themselves as YAC, if the care receiver was from the family, they defined themselves solely with their family relationship (e.g., child, grandchild, sibling; Lewis, 2017). Moreover, giving a definition of caring at the beginning of research did not lead young people to identify themselves as YAC. This non-identification was not gendered and could be related to the fact that caring was seen as a familial obligation or a normal responsibility rather than a distinct role (Struckmeyer, 2013). However, some YAC preferred not to be labeled as carers because of stigma and fear of mistreatment. They adjusted their ways of introducing themselves in relation to the time, setting, and audience. In doing this, these YAC partially accepted the identity (Lewis, 2017). In addition, a YAC who was the primary caregiver or who provided care for both a parent and a sibling was more likely to self-identify as a carer than a YAC who shared the caring responsibilities or who cared for siblings only (Lewis, 2017). For American YAC, being a carer was seen as adding value to their life (Lewis, 2017). In general, when YAC understood that the care receiver's health condition was a fixture of their life and that the care receiver was fallible (i.e., could die), YAC assimilated their caring role and identity (Thompson et al., 2017). Above all, in order to identify themselves as carers, YAC needed their family to recognize their contributions as carers (Lewis, 2017).

#### Living arrangement

A majority of YAC were co-resident with the care receiver (Becker and Becker, 2008a,b), unless they were students (Haugland et al., 2020). Those who left home to attend higher education or to live with a partner could go back each weekend to do instrumental caring activities (Becker and Becker, 2008a). YAC may choose not to leave home because of close family ties or concern about leaving the care receiver alone, or to prevent greater caring responsibilities from falling on a sibling or other family member (Becker and Becker, 2008a,b; Hamilton and Adamson, 2013). Decisions to leave home depended on finances, the care receiver's type of illness or disability, caring responsibilities (i.e., extent, severity and intensity of the needs expressed now and anticipated for the future), the existence of other support for the care receiver, and pressure or encouragement from the care receiver (Becker and Becker, 2008a; Hamilton and Adamson, 2013). In many cases, YAC had no choice (Becker and Becker, 2008a). However, living independently was a central preoccupation of YAC (Hamilton and Adamson, 2013). Living away from home enabled YAC to increase their physical and emotional independence (Becker and Becker, 2008a).

#### Physical, psychological, and adaptative outcomes

Physical and psychological outcomes of YAC responsibilities could be both negative and positive. The negative health effects of a caring role manifested physically and psychologically. YAC reported poor physical health that included fatigue, exhaustion, insomnia, extra weight, backaches, depression, and a tendency toward colds and ulcers (Mancini et al., 2006; Becker and Becker, 2008a; Cass et al., 2011; Hamilton and Adamson, 2013; Haugland et al., 2020). This impact intensified over the caregiving years (Cass et al., 2011). More precisely, a dose-response relationship was found between the somatic symptom burden and the amount of care responsibilities (Haugland et al., 2020). YAC appeared to develop more physical health concerns in relation to intense and prolonged periods of caring (Hamilton and Adamson, 2013). In fact, YAC often neglected their own health in favor of the health of the care receiver (Becker and Becker, 2008a) and frequently became sick (Hamilton and Adamson, 2013). Some YAC also engaged in risk-taking behaviors like consuming drugs and alcohol or having unsafe sex (Becker and Becker, 2008a; Grenard et al., 2020). These risk-taking behaviors could be considered as a form of “escapism” due to the pressure of caring roles, strained family relationships, and loneliness (Becker and Becker, 2008a). Some risk-taking behaviors seemed more represented than others. For example, cigarette smoking was linked to caregiving and expecting to become a caregiver within the next two years, unlike drinking and e-cigarette use (Grenard et al., 2020). Regarding their overall health, when YAC had congenital illness, learning difficulties, or sports injuries, caring tasks were more difficult, tiring, and stressful (Becker and Becker, 2008a). Interestingly, there were more physical health consequences of caregiving for YAC than for YC (Cass et al., 2011).

The negative impact extended to psychological states. YAC presented worry, stress, anxiety, depression, anger, upset, resentment, loneliness, and resignation (Becker and Becker, 2008a; Cass et al., 2011; Hamilton and Adamson, 2013; Greene et al., 2017; Grenard et al., 2020; Haugland et al., 2020). Psychological discomfort may stem from YAC perceiving themselves as different from other young adults (Jones, 2018). Compared to non-carers, YAC students presented higher rates of depressive and anxious traits (Greene et al., 2017; Haugland et al., 2020). YAC students' levels of affective symptomatology were clinically significant. They appeared more vulnerable to psychiatric distress, considering the burden of caregiving and the academic pressure (Greene et al., 2017). Among youth, frequent mental distress was 50% higher in YAC than in non-carers (Grenard et al., 2020). Although caring activities were not associated with psychological effect (Struckmeyer, 2013), frequent mental distress had been linked to the type of caring activities and the weekly hours of care provided (Grenard et al., 2020; Haugland et al., 2020). YAC thus reported higher distress than others when they provided personal tasks only or personal tasks combined with household tasks (Grenard et al., 2020). Concerning the weekly hours of care, providing < 19 h or more than 40 h of care per week led to a similar impact on frequent mental distress (Grenard et al., 2020). Even caring for a small amount of time was a risk factor for mental distress (Grenard et al., 2020; Haugland et al., 2020). Among students, there was no difference between YAC and non-carers regarding self-esteem (Greene et al., 2017). However, YAC had lower scores of life satisfaction than non-carers (Haugland et al., 2020). A higher amount of caring activities was associated with lower life satisfaction (Haugland et al., 2020).

Caring also had positive outcomes for YAC. The caring role led YAC to develop positive psychosocial attributes like empathy and understanding (Becker and Becker, 2008a,b). Additionally, YAC appeared to be more sensitive and respectful and less judgmental than other young adults (Becker and Becker, 2008a; Cass et al., 2011).

Having a caring role could accelerate YAC maturity and sense of self (Becker and Becker, 2008a; Cass et al., 2011; Jones, 2018). Their responsibilities led them to develop skills and strategies that helped them cope with crisis or complex situations (Becker and Becker, 2008a). In addition, YAC developed specific adulthood-related skills like managing home care, cooking, and officialdom (Becker and Becker, 2008a). YAC thus felt competent and effective due to their specific caring skills and knowledge (Jones, 2018). They believed that they were able to look after the care receiver as well as themselves, which had a positive effect on their ability to be resilient, flexible, and strong (Jones, 2018).

Regarding YAC adaptative outcomes, no studies investigated coping strategies in the same way. Among student samples, one study showed no difference between YAC and non-carers (Greene et al., 2017), whereas another found that YAC showed more emotion-focused strategies (Boumans and Dorant, 2018). YAC appeared to be emotionally mature and more able to control their feeling states (Jones, 2018). The main coping strategies identified among YAC were establishing a routine or schedule, separating their home and school lives, finding humor in difficult situations, accepting things as they were, finding the positive aspects of a situation and making sure they had personal time and space (Cass et al., 2011). Other coping strategies highlighted were praying, talking to family and friends, and using the internet (Levine et al., 2005).

#### Interpersonal relationships

Having a caring role led YAC to develop a closer relationship with the care receiver, as YAC felt useful and able to make the receiver feel better (Becker and Becker, 2008a; Cass et al., 2011; Thompson et al., 2017). When the care receiver was a grandparent, YAC had a generally positive perception of her/him related to the quality of contact in the caregiving relationships (Canell and Caskie, 2019). The caregiving relationships could be seen as the “best things” about being a carer (Cass et al., 2011). However, the relationships were damaged if caring responsibilities had negatively affected YAC in their studies or employment (Becker and Becker, 2008a). Because of their developmental stage of emerging adulthood, YAC needed to experience independence from their families. The lack of this opportunity could lead to strained relationships with the care receiver, especially if he/she was a parent (Becker and Becker, 2008a). YAC were more likely than YC to comment and offer a reflection about how their parent-child relationships changed over time (Cass et al., 2011).

Caring responsibilities left YAC with the feeling that they had insufficient time for themselves and for social and leisure activities (Becker and Becker, 2008a,b; Cass et al., 2011). Indeed, it seemed difficult for YAC to concentrate on their social life due to worries about the care receiver (Leu et al., 2018). Compared to non-carers, YAC were less likely to engage in extracurricular activities (Greene et al., 2017). It appeared that having a caring role had greater consequences for the social lives of YAC than of YC (Cass et al., 2011). The effects on social life were consistent whether YAC undertook a caring role for many years had or became carers more recently; there was no influence from the length of time in caring roles (Hamilton and Adamson, 2013). Being a carer could be a challenge when making and keeping friends, as YAC could experience strain and difficulty within their relationships (Becker and Becker, 2008a). Talking about the caring role could have positive or negative impacts on their relationships with peers (Leu et al., 2018). When YAC did not want to talk about their caring role, they avoided social interaction (Leu et al., 2018). For some YAC, the high maturity fostered by their caring role influenced their capacity to make friends because they were not carefree and felt different from their peers (Becker and Becker, 2008a). Although the university context offered a chance to socialize more than other school levels (Becker and Becker, 2008a), YAC students rarely socialized outside scheduled class hours and feared being stigmatized as inferior or incompetent because of their caring status (Day, 2019). However, it appeared easier for YAC students to have leisure time and socialize than for YAC who were not students (Becker and Becker, 2008a). YAC often chose friends who understood their situation (Becker and Becker, 2008a) and who may also be carers, which led to a feeling of being supported (Leu et al., 2018).

#### Education and employment

YAC could be enrolled in higher education as well as employed or not in education, employment, or training (NEET). However, YAC were less likely to be students and more likely to be unemployed than non-carers (Grenard et al., 2020). Being a YAC for many years led to a similar experience of education and employment as being a YAC for a few months (Hamilton and Adamson, 2013).

Regarding education and their past school experience, YAC could experience school as positive when caring responsibilities were shared with other family members or when the school staff was understanding (Becker and Becker, 2008a). However, having a caring role could cause YAC to be late or absent or to fail to complete homework in time (Becker and Becker, 2008a). YAC could also express negative outcomes from their school experience, such as a lack of understanding from school staff or bullying from peers because they were too mature or because their family was seen as different. These negative outcomes could lead to low academic achievement and poor attendance (Becker and Becker, 2008a). In contrast to the school context, higher education provided some YAC with a more positive experience because of its greater flexibility, adult-oriented focus, and more understanding and supportive staff (Becker and Becker, 2008a; Cass et al., 2011). Nevertheless, some YAC frequently missed classes (Mancini et al., 2006) or were forced to leave higher education prematurely because of the competing demands of caring and studying, the increased workload, or a feeling of being unsupported regarding their caring situation (Becker and Becker, 2008a; Cass et al., 2011; Hamilton and Adamson, 2013). Indeed, YAC had to balance caregiving and studying (Becker and Becker, 2008a; Cass et al., 2011; Leu et al., 2018; Day, 2019). They could be divided between the desire to be a good student and to be a good carer (Kettell, 2018). As in the school context, university academic achievement and progression were facilitated by encouragement and support from parents or significant others (Becker and Becker, 2008a). Some YAC preferred to stay at home to “keep an eye” on the care receiver; they then spent a minimal amount of time on campus and did not go to social events (Mancini et al., 2006; Day, 2019). These YAC spent more time studying, and therefore for some of them, there was a modest positive impact of caregiving on their educational achievement (Mancini et al., 2006). Nevertheless, some YAC experienced challenges in maintaining study routines, keeping up to date with coursework, and investing quality time and effort in their homework (Day, 2019). YAC could then feel less satisfied with their academic performance and achievement than other young adults, as they thought they should do better (Day, 2019).

Regarding vocational and career choices, for some YAC students, their caring role directly influenced these paths. This influence could be conscious or unconscious and lead YAC to choose a care-related career or not to (Becker and Becker, 2008a). It appeared that there were more YAC in vocational education like nursing than non-carers (Boumans and Dorant, 2018). YAC decisions about university majors and career plans could be directly related to parental illness (e.g., becoming a nutritionist when a parent had diabetes; Thompson et al., 2017). However, YAC vocational aspirations could also be restrained by the realities of balancing their ambitions with their caring role (Hamilton and Adamson, 2013; Kettell, 2018). For example, YAC could choose a local higher education institution to stay near their care receiver and continue to fulfill their caring role. In that sense, caring responsibilities shaped YAC institution choices (Becker and Becker, 2008a; Cass et al., 2011; Hamilton and Adamson, 2013).

Many YAC experienced financial hardship because of caring and living in low-income families (Becker and Becker, 2008a; Grenard et al., 2020; Haugland et al., 2020). This situation could lead to dropping out of higher education to take a full-time job, or taking a part-time job in addition to their studies. However, YAC students did not often hold part-time jobs because of the difficulty of balancing education, work, and caring roles (Becker and Becker, 2008a).

Some YAC felt that they were less prepared than other young adults to enter the workforce and less likely to succeed in securing employment (Day, 2019). It could be difficult to combine work with the caring role (Cass et al., 2011; Hamilton and Adamson, 2013). Employment was thus a core issue for YAC (Hamilton and Adamson, 2013). YAC expressed the need to have flexible and adaptable work as well as a “good employer,” which meant a comprehensive one (Cass et al., 2011; Hamilton and Adamson, 2013). Therefore, YAC caring responsibilities could constrain their employment choice, as a job had to be perceived as suitable in relation to caring tasks (Hamilton and Adamson, 2013). Some YAC could be temporarily in the NEET category because of the difficulty in finding a first job due to lacking qualifications, experience, respite care arrangements, social skills, self-confidence, or time (Becker and Becker, 2008a). Overall, YAC seemed to clearly understand their caring role and how it affected their education, employment, and future life opportunities (Cass et al., 2011).

### How to support YAC

#### Needs for support

YAC had the same needs as any young adults, although they also had specific needs related to their caring responsibilities, their experience, and their identity as carers (Becker and Becker, 2008a). These specific needs depended on family circumstances, the care receiver's illness/disability and needs, financial outcomes, and availability of other carers in or out of the family and could be increased by the amount of caring (Becker and Becker, 2008b). Regarding educational and employment contexts, it appeared that YAC needed to improve awareness about the problems posed by their circumstances (Cass et al., 2011). Support and understanding from university staff and student counselors could be highly significant for some YAC students (Becker and Becker, 2008a). It could be positive for them that university staff knew about their caring role (Cass et al., 2011). YAC students seemed to need assistance concerning several subjects in particular, such as student finance and counseling. Indeed, as YAC did not see the end to their caring responsibilities, they could be unable to have long-term aspirations or a clear career choice. They could need to be supported in relation to their vocational exploration and decisions (Becker and Becker, 2008a). A complicating factor is that when a YAC missed school, they could also miss career events and advice organized by the school (Becker and Becker, 2008a). YAC who were NEET could need support, advice, and information about potential jobs and training (Becker and Becker, 2008a). In general, YAC had needs for information, advice, and guidance (e.g., grants, healthy diet, wellbeing, caring); services and support (e.g., counseling, breaks from caring, contingency planning, public transport, housing); education and training (e.g., guidance, opportunities); activities and peer support (e.g., leisure activities, social networking, social inclusion); and job-seeking support and flexible employment (e.g., career guidance, labor market participation; Becker and Becker, 2008a). Above all, YAC needed communication during and around their caring situations. This communication could be within the family, with the extended family, with professionals, and with peers (Leu et al., 2018). YAC needed to “understand” as something they both had (i.e., knowledge and acceptance of the situation) and communicated (Thompson et al., 2017). To fulfill this need, some YAC could express the desire to participate in community events to talk about their situations using an educational approach (Jones, 2018).

Concerning support services, many included studies agreed that YAC needed a specific service (Becker and Becker, 2008a; Hamilton and Adamson, 2013; Day, 2015a; Kent, 2020). If YAC were not recognized and considered, they could be ignored or remain invisible to health, social, and carer services (Becker and Becker, 2008a). Most support services for young carers were not relevant to people over 18 years old. YAC who had had caring responsibilities since childhood or adolescence might be involved in support services, but when they reached 18, this support ended and nothing else was proposed (Becker and Becker, 2008a,b). There was then a clear gap in service provision when young carers became adults (Becker and Becker, 2008b; Hamilton and Adamson, 2013). Moreover, adult support services did not match with YAC needs, as they were generally used by and promoted for older carers (i.e., 40 years and over; Becker and Becker, 2008b). For YAC becoming carers between 18 and 25 years, they were in an in-between period, and no appropriate services were available (Becker and Becker, 2008b). However, it appeared that most YAC did not know that they had the right to access support services even in an educational context (Becker and Becker, 2008a,b). Even if YAC knew about support services, these could remain less accessible because of the time required alongside caring responsibilities (Hamilton and Adamson, 2013) or because of sociocultural and caring characteristics. In the United States, being identified as YAC did not necessarily lead to an opportunity to enter support services, unlike in the United Kingdom (Lewis, 2017). Concerning caring characteristics, if the care receiver was a sibling, YAC were less likely to engage in support services (Lewis, 2017). Being engaged in support services did not mean that YAC self-identified themselves as YAC, but only that they needed support (Lewis, 2017).

Regarding the care receiver, YAC often identified and addressed unmet needs (Becker and Becker, 2008a). Some YAC reported having difficulties obtaining medical and nonmedical assistance for the care receiver (Levine et al., 2005). Indeed, YAC could be unaware of what support might be available for the care receiver (Becker and Becker, 2008a,b). These results revealed that one need of YAC was for the care receiver to be better supported by health and social services. Thus, a way to help YAC was to support the care receiver (Becker and Becker, 2008b; Day, 2015a; Leu et al., 2018; Joseph et al., 2020).

#### Available support services

The included studies all reported that few support services were devoted to YAC. Becker and Becker (2008a) found that in the United Kingdom, only one service was specifically designed for YAC (i.e., Action for Young Carers plus) and four were for young carers between 16 and 25 years without age distinction (i.e., York Carers Center, Youth Action Wiltshire, Islington Young Adult Carers Group, Hub Young Carers). These projects were developed in order to improve YAC mental and physical wellbeing; enhance YAC self-confidence and self-esteem; promote age-appropriate respite activities; assist YAC in accessing employment, training, or education; ensure income/benefit maximization; understand and promote choices; provide access to training, and sometimes certification, on aspects of caring and life skills; provide access to assessment and additional support services where appropriate. However, the provision of support services was more available in urban areas than in rural ones (Becker and Becker, 2008b). Outside the United Kingdom, a lack of support services infrastructure was found in Australia (Hamilton and Adamson, 2013; Day, 2015a) as well as the United States (Kent, 2020). This lack of support was more significant in some countries than in others, depending on their local policy and their awareness of YAC circumstances (Joseph et al., 2020).

#### Recommendations for support services

The first step that could lead to better support for YAC is identification. Improving YAC identification in higher education, workplace, health or social care settings should improve formal assessment of their needs (Becker and Becker, 2008b). Being involved in support services could also solidify YAC self-identification as carers (Lewis, 2017). In order to improve services' identification, Joseph et al. (2020) proposed conceptualizing caring on three levels: “caring about,” which referred to YAC who help in a minimal way (e.g., household chores), not much more than many non-carers; “caring for,” which referred to YAC who take on a level of responsibility (e.g., household chores, nursing duties) but not to the point of interfering with their social and educational lives; and “need care,” which referred to YAC who take on a high level of responsibility (e.g., household chores, nursing duties, intimate care, emotional care) beyond that of non-carers, and who are enabled to engage in social and education lives. These three levels should facilitate the development of more adapted interventions and supports. Once YAC are clearly identified, support services should help them to navigate through important transitional periods, such as from childhood to adulthood, from high school to higher education, or from education to employment (Hamilton and Adamson, 2013). In other words, support services should be congruent with the realities of YAC and consider that they are emerging adults (Kent, 2020). Furthermore, support services should promote the de-stigmatization of illness, disability, and caring in order to reduce the negative reaction YAC may face regarding their situation (Lewis, 2017). This highlights the need to communicate during and around caring situations and also emphasizes that support services should involve the whole family (Hamilton and Adamson, 2013; Leu et al., 2018; Kent, 2020).

In an educational context, university staff should be more informed about YAC problems (Becker and Becker, 2008a) as well as about available support services (Kettell, 2018). More information could lead to more flexible homework arrangements, connections with other health or social services, more appropriate YAC identification, encouragement of career choice exploration and consideration, and measures to decrease dropping out (Becker and Becker, 2008a; Hamilton and Adamson, 2013; Kettell, 2018). To this end, Kettell (2018) proposed a “carers' passport” that would signify to all university staff that a student is also a carer. This passport could be a visible document, like a card or a booklet, that would prevent YAC from having to explain their circumstance multiple times and would come with priority access (e.g., car parking). However, this passport would require that YAC consent to publicly talk about and reveal their carer status (Kettell, 2018). Another solution proposed was that one person from the university staff could be clearly identified as a resource for YAC. Then, YAC could meet this resource person to discuss their needs and difficulties (Kettell, 2018). To support peer relationships, universities should promote the establishment of YAC student associations or societies. This kind of social group should give opportunities for socialization, reduce isolation, and provide a sense of belonging through social events and online forums (Kettell, 2018). In addition to associations or societies, universities could provide peer mentors during the first semester to help YAC adapt to and stay in higher education (Kettell, 2018).

Regarding YAC health behavior, support services should encourage health-promoting behaviors (e.g., exercise, socialization) and discourage adverse health behaviors (e.g., drinking, smoking) to regulate stress responses (Kent, 2020). In addition, support services should also boost functional coping strategies, such as problem-focused strategies, to simultaneously enhance healthier behaviors (Kent, 2020). Overall, Becker and Becker (2008a) gave eight recommendations: (1) clearly identify the outcomes of any intervention regarding support services objectives and resources as well as YAC needs; (2) include YAC in the discussion and planning of interventions; (3) involve local workers from services for YC, adult social care, and adult carers in the intervention development; (4) develop partnerships between YC projects and care centers in order to share learning, materials, and ideas; (5) involve institutions and organizations (e.g., university, employers) in identifying and engaging with YAC; (6) be more aware of YAC-specific needs and the way organizations and support services deliver services to them in particular; (7) provide information about YAC legal rights at the local level; and (8) integrate YAC needs and intervention outcomes into the local authority carer's strategy.

## Discussion

Since the first publication on the subject (Levine et al., 2005) and the formal definition (Becker and Becker, 2008a), YAC have become a growing body of interest. The purpose of the present study was to gain insights into the YAC population using systematic review methodology. We highlighted that among the literature, only 23 studies of varying quality clearly addressed YAC as informal carers aged 18–25. Our results showed different ways to identify YAC in research. Thus, the prevalence reported is quite heterogeneous. Our findings revealed the distinct traits of the YAC population in light of emerging adulthood and the need to develop specific support services for them.

### YAC identification and prevalence

One of the main findings of the present systematic review is that identifying and assessing YAC is a major problem (Becker and Becker, 2008b). Our research shows the varied procedures for YAC identification and, in results, the heterogeneity of prevalence. Three identification procedures were used in the studies: YAC already identified as carers, YAC self-identification and criteria based on wide definitions. Being already identified implies that YAC may be involved in support services or associations. However, our findings show that there are most often no appropriate services (Becker and Becker, 2008a; Hamilton and Adamson, 2013; Day, 2015a; Kent, 2020) and that YAC could find themselves without support (Becker and Becker, 2008b; Hamilton and Adamson, 2013). In other ways, identifying YAC through support services can lead to an underestimation of their prevalence.

The use of a self-identification procedure could also lead to underestimation. For most YAC, self-identification is dependent on how others see them. Those could be health or social professionals as well as family members (Becker and Becker, 2008a; Lewis, 2017). Moreover, some YAC may prefer to hide their carer identity due to fear of stigma. This fear seems to be culturally sensitive. For example, for Americans, caring responsibilities is an added value (Lewis, 2017). It could be presumed that differences in prevalence between two countries could be partially explained by cultural context. It is worth noting that even if YAC are involved in support services, they do not always identify themselves as carers (Lewis, 2017).

The third identification procedure was based on a wide definition. This definition is based on Becker and Becker's (2008a) and could include the nature of support, its frequency, the affiliation with the care receiver, and the care receiver's type of illness or disability. The fact that only one study included these four notions (Grenard et al., 2020) highlighted that for the majority of the studies, a partial definition was sufficient. However, literature has shown that the nature and frequency of daily living activities allows young carers to be differentiated from non-carers (Warren, 2005) and that having an ill or disabled relative does not necessarily lead to becoming a carer (Blanc, 2010).

A complete definition is needed to ensure that YAC are accurately identified. Moreover, for studies based on census information (Becker and Becker, 2008a,b), the adults in the household were questioned, and those could be the YAC or not. As caring responsibilities can be normalized by the family due to the YAC age and stage of life (Becker and Becker, 2008a,b; Cass et al., 2011; Lewis, 2017), it is possible that identifying YAC through adult responses can lead to misidentification. This misidentification could explain the gap between prevalence among youth in the United Kingdom, which is based on a census, and in the United States, which is not (5.30 vs. 18.10%). Above all, the present systematic review underlines the need to develop and propose a universal identification procedure in order to estimate YAC prevalence among countries and favor cultural comparison. This procedure should be based on a complete definition (i.e., nature of support, frequency, affiliation with the care receiver, and care receiver type of illness or disability) and on Joseph et al. (2020) proposition to designate three levels of caring: “caring about,” “caring for,” and “need care.” One way to develop this procedure could be to consider the nature and amount of caring responsibilities, as had been proposed among young carers (Nagl-Cupal et al., 2014; Untas et al., 2022). One validated self-reported questionnaire may be used: the Multidimensional Assessment of Caring Activities for Young Carers (Joseph et al., 2009, 2019). This questionnaire seems to be a relevant screening tool for identifying YAC among the general population and highlighting the differences between normal helping activities and caring ones (Chevrier et al., 2022a). One other validated self-reported questionnaire that may be used is the Youth Activities of Caregiving Scale (Ireland and Pakenham, 2010). Overall, future prevalence research should propose a comparison of the different identification procedures highlighted in this systematic review in order to investigate their differences and to ensure a clear estimation of YAC.

### YAC characteristics and supports

Using a narrative method (Baumeister and Leary, 1997), the present systematic review showed nine themes under YAC characteristics: (1) ways into caring; (2) care receiver; (3) caring responsibilities; (4) amount of caring; (5) self-identification as a carer; (6) living arrangement; (7) physical, psychological, and adaptative outcomes; (8) interpersonal relationships; and (9) education and employment. Our findings show that there is a variety of caring experience. The care receiver can be from the nuclear family or a more distant relationship, like a neighbor, and can suffer from chronic disease or disability as well as mental disorder. The caring responsibilities are activities of daily living and instrumental activities of daily living, and the amount of caring varies from part-time to full-time responsibility (13–50 h per week). Self-identification as a carer can be embraced or can be rejected due to fear of stigma. The living arrangement can be co-resident as well as living independently; both physical and psychological outcomes can be either negative or positive; the relationships with the care receiver can be straightened or not; and the education experience can be affected or not. These results point out that YAC are not a unified population. Each YAC have his/her one issues. For example, caring for an ill/disabled parent is associated with poorer mental health than caring for another ill/disabled family member (Landi et al., 2022). Future research should consider YAC' specificities, such as who the care receiver is, to better understand and capture their experience and its consequences.

According to Joseph et al. (2020), the term “young adult carers” is a broad descriptor only. Future research should investigate YAC characteristics using a person-oriented approach, like in Chevrier et al. (2022b), in order to identify individual differences and to specify subgroups' characteristics. Nevertheless, one caring activity seems to stand out: emotional support. The literature agrees that this activity is a core issue for YAC (Becker and Becker, 2008a; Day, 2015a; Boumans and Dorant, 2018; Chevrier et al., 2022b). It is a “caring at a distance” task (Becker and Becker, 2008a) that can be fulfilled even if YAC do not live with their care receiver (Chevrier et al., 2022b).

Furthermore, our results underline that caring responsibilities shape YAC educational choices (Becker and Becker, 2008a; Cass et al., 2011; Hamilton and Adamson, 2013; Grenard et al., 2020; Haugland et al., 2020) and then vocational choices. Through a counseling psychology lens, families can strain vocational decisions (Brasselet and Guerrien, 2019) and major life events, like endorsing a caring role, as well as contribute to identity construction and career decisions (Savickas and Pouyaud, 2016). Future research should investigate in more detail YAC educational choices regarding their identity construction and vocational decisions. Finally, our findings also demonstrate that independence is a central preoccupation of YAC (Becker and Becker, 2008a; Hamilton and Adamson, 2013), as it is for any emerging adults (Arnett, 2001). Independence, as an expression of autonomy, is defined as the capacity to make decisions by oneself, independently from parents and family (van Petegem et al., 2012). Being independent is a criterion for reaching adulthood (Arnett, 2011). Considering YAC as emerging adults leads to rethinking research in the light of a developmental approach.

The present systematic review highlights that YAC have several needs for support. These needs are common with those of emerging adults (e.g., finance, career choice, jobs and training) but also specific to their caring situation. Beyond the need for information about available support services, it appears that YAC also need to communicate and understand about their caring experience (Thompson et al., 2017; Jones, 2018; Leu et al., 2018). While YAC needs have been highlighted in the literature, all included studies agreed that there are no available support services clearly devoted to this population. This lack of services reflects the low level of policy awareness about this problem (Joseph et al., 2020). Nevertheless, our findings point out practical recommendations for the policy as well as educational levels. The first, and more important, recommendation is to improve YAC identification. The second is to consider YAC as emerging adults and then as young people facing many life transitions (e.g., education to employment). The third is to communicate during and around the caring experience. Kent (2020) and Kettell (2018) proposed several solutions within and out of the educational context (e.g., boosting functional coping strategies, “carers' passport”). Future research should evaluate the effect of these solutions in order to disseminate them.

### Limitations

This systematic review has several limitations. First, the present results should be read with caution, given the characteristics of the included studies. Most were from gray literature and not peer reviewed (*n* = 11), gender biased (*n* = 15), and with fewer than 100 participants (*n* = 10). Some were only on YAC (*n* = 6), on YAC students (*n* = 6), or on female YAC (*n* = 3). All studies included were cross-sectional. The evolution and development of YAC over time have not yet been explored. Numerous studies have low to moderate quality due to a lack of precision. It could also be presumed that the Crowe Critical Appraisal Tool evaluation method used did not best suit literature reviews or letters designs. Moreover, it is also worth noting that the included studies took place in North American, European or Australian contexts. The specificity of YAC populations outside of these areas is still unknown.

The second limitation is that as the present systematic review highlighted, there is no unique YAC identification procedure, so some excluded studies might have included YAC among their samples. The wide variation of the definition used to refer to YAC might have excluded relevant studies. Third, as emerging adulthood could extend up to age 29 (Arnett, 2014; Arnett et al., 2014) regarding cultural expectations (Jensen, 2011), considering YAC aged 18–25 years in this research might have led to excluding some relevant studies. Finally, the present systematic review addressed YAC problems in a general way. This is a first step; future works need to investigate more specific questions in order to highlight the particularities of YAC in a given area (e.g., their way into caring, physical and psychological outcomes, employment situations, and NEET status).

## Conclusion

The present systematic review highlights the importance of taking interest in the YAC population in both research and practice fields. It points out that one universal identification procedure should be established and that YAC should be considered from a developmental perspective as emerging adults. However, the diversity of YAC caring experiences highlights the need to better investigate individual differences and subgroup specificities. In order to improve our awareness of YAC situations, future research should consider a person-oriented approach and more precisely investigate YAC characteristics from this perspective.

## Data availability statement

The raw data supporting the conclusions of this article will be made available by the authors, without undue reservation.

## Author contributions

AU, GD, and BC contributed to conception and design of the study. BC organized the database and wrote the first draft of the manuscript. BC and KL performed the statistical analysis. All authors contributed to manuscript revision, read, and approved the submitted version.

## Funding

This work was supported by Klésia. The funder had no role in the study design, collection, analysis, interpretation of the data, writing of the manuscript, or the decision to submit the paper for publication.

## Conflict of interest

The authors declare that the research was conducted in the absence of any commercial or financial relationships that could be construed as a potential conflict of interest.

## Publisher's note

All claims expressed in this article are solely those of the authors and do not necessarily represent those of their affiliated organizations, or those of the publisher, the editors and the reviewers. Any product that may be evaluated in this article, or claim that may be made by its manufacturer, is not guaranteed or endorsed by the publisher.
