# Supplementary material for: Young adult carers' identification, characteristics, and support: A systematic review
Source: Front Psychol. 2022 Oct 24;13:990257. doi: 10.3389/fpsyg.2022.990257 (PMC9639750; doi:10.3389/fpsyg.2022.990257)
Supplement: Supplementary file 1 [file Data_Sheet_1.docx]

**Supplemental Material 1: PRISMA Main and Abstract Checklists**

# PRISMA 2020 Main Checklist

| Topic | No. | Item | Location where item is reported |
| --- | --- | --- | --- |
| **TITLE** |  |  |  |
| **Title** | 1 | Identify the report as a systematic review. | Title |
| **ABSTRACT** |  |  |  |
| **Abstract** | 2 | See the PRISMA 2020 for Abstracts checklist |  |
| **INTRODUCTION** |  |  |  |
| **Rationale** | 3 | Describe the rationale for the review in the context of existing knowledge. | p. 1-2 |
| **Objectives** | 4 | Provide an explicit statement of the objective(s) or question(s) the review addresses. | p. 2 |
| **METHODS** |  |  |  |
| **Eligibility criteria** | 5 | Specify the inclusion and exclusion criteria for the review and how studies were grouped for the syntheses. | p. 2 |
| **Information sources** | 6 | Specify all databases, registers, websites, organisations, reference lists and other sources searched or consulted to identify studies. Specify the date when each source was last searched or consulted. | p. 2 |
| **Search strategy** | 7 | Present the full search strategies for all databases, registers, and websites, including any filters and limits used. | p. 2 |
| **Selection process** | 8 | Specify the methods used to decide whether a study met the inclusion criteria of the review, including how many reviewers screened each record and each report retrieved, whether they worked independently, and if applicable, details of automation tools used in the process. | p. 2 |
| **Data collection process** | 9 | Specify the methods used to collect data from reports, including how many reviewers collected data from each report, whether they worked independently, any processes for obtaining or confirming data from study investigators, and if applicable, details of automation tools used in the process. | p. 2-3 |
| **Data items** | 10a | List and define all outcomes for which data were sought. Specify whether all results that were compatible with each outcome domain in each study were sought (e.g. for all measures, time points, analyses), and if not, the methods used to decide which results to collect. | p. 3 |
|  | 10b | List and define all other variables for which data were sought (e.g. participant and intervention characteristics, funding sources). Describe any assumptions made about any missing or unclear information. | Not applicable |
| **Study risk of bias assessment** | 11 | Specify the methods used to assess risk of bias in the included studies, including details of the tool(s) used, how many reviewers assessed each study and whether they worked independently, and if applicable, details of automation tools used in the process. | p. 3 |
| **Effect measures** | 12 | Specify for each outcome the effect measure(s) (e.g. risk ratio, mean difference) used in the synthesis or presentation of results. | Not applicable |
| **Synthesis methods** | 13a | Describe the processes used to decide which studies were eligible for each synthesis (e.g. tabulating the study intervention characteristics and comparing against the planned groups for each synthesis (item 5)). | Not applicable |
|  | 13b | Describe any methods required to prepare the data for presentation or synthesis, such as handling of missing summary statistics, or data conversions. | Not applicable |
|  | 13c | Describe any methods used to tabulate or visually display results of individual studies and syntheses. | Not applicable |
|  | 13d | Describe any methods used to synthesize results and provide a rationale for the choice(s). If meta-analysis was performed, describe the model(s), method(s) to identify the presence and extent of statistical heterogeneity, and software package(s) used. | p. 3 |
|  | 13e | Describe any methods used to explore possible causes of heterogeneity among study results (e.g. subgroup analysis, meta-regression). | Not applicable |
|  | 13f | Describe any sensitivity analyses conducted to assess robustness of the synthesized results. | Not applicable |
| **Reporting bias assessment** | 14 | Describe any methods used to assess risk of bias due to missing results in a synthesis (arising from reporting biases). | Not applicable |
| **Certainty assessment** | 15 | Describe any methods used to assess certainty (or confidence) in the body of evidence for an outcome. | Not applicable |
| **RESULTS** |  |  |  |
| **Study selection** | 16a | Describe the results of the search and selection process, from the number of records identified in the search to the number of studies included in the review, ideally using a flow diagram. | p. 3 and Figure 1 |
|  | 16b | Cite studies that might appear to meet the inclusion criteria, but which were excluded, and explain why they were excluded. | p.3 |
| **Study characteristics** | 17 | Cite each included study and present its characteristics. | p. 3 |
| **Risk of bias in studies** | 18 | Present assessments of risk of bias for each included study. | p. 3 |
| **Results of individual studies** | 19 | For all outcomes, present, for each study: (a) summary statistics for each group (where appropriate) and (b) an effect estimate and its precision (e.g. confidence/credible interval), ideally using structured tables or plots. | Table 1 and Supplemental Material 3 |
| **Results of syntheses** | 20a | For each synthesis, briefly summarise the characteristics and risk of bias among contributing studies. | Not applicable |
|  | 20b | Present results of all statistical syntheses conducted. If meta-analysis was done, present for each the summary estimate and its precision (e.g. confidence/credible interval) and measures of statistical heterogeneity. If comparing groups, describe the direction of the effect. | Not applicable |
|  | 20c | Present results of all investigations of possible causes of heterogeneity among study results. | Not applicable |
|  | 20d | Present results of all sensitivity analyses conducted to assess the robustness of the synthesized results. | Not applicable |
| **Reporting biases** | 21 | Present assessments of risk of bias due to missing results (arising from reporting biases) for each synthesis assessed. | Not applicable |
| **Certainty of evidence** | 22 | Present assessments of certainty (or confidence) in the body of evidence for each outcome assessed. | Not applicable |
| **DISCUSSION** |  |  |  |
| **Discussion** | 23a | Provide a general interpretation of the results in the context of other evidence. | p. 13-15 |
|  | 23b | Discuss any limitations of the evidence included in the review. | p. 15 |
|  | 23c | Discuss any limitations of the review processes used. | p. 15 |
|  | 23d | Discuss implications of the results for practice, policy, and future research. | p. 13-15 |
| **OTHER INFORMATION** |  |  |  |
| **Registration and protocol** | 24a | Provide registration information for the review, including register name and registration number, or state that the review was not registered. | p. 2 |
|  | 24b | Indicate where the review protocol can be accessed, or state that a protocol was not prepared. | p. 2 |
|  | 24c | Describe and explain any amendments to information provided at registration or in the protocol. | Not applicable |
| **Support** | 25 | Describe sources of financial or non-financial support for the review, and the role of the funders or sponsors in the review. | Title page |
| **Competing interests** | 26 | Declare any competing interests of review authors. | p. 16 |
| **Availability of data, code, and other materials** | 27 | Report which of the following are publicly available and where they can be found: template data collection forms; data extracted from included studies; data used for all analyses; analytic code; any other materials used in the review. | Not applicable |

#####

# PRIMSA Abstract Checklist

| Topic | No. | Item | Reported? |
| --- | --- | --- | --- |
| **TITLE** |  |  |  |
| **Title** | 1 | Identify the report as a systematic review. | Yes |
| **BACKGROUND** |  |  |  |
| **Objectives** | 2 | Provide an explicit statement of the main objective(s) or question(s) the review addresses. | Yes |
| **METHODS** |  |  |  |
| **Eligibility criteria** | 3 | Specify the inclusion and exclusion criteria for the review. | Yes |
| **Information sources** | 4 | Specify the information sources (e.g. databases, registers) used to identify studies and the date when each was last searched. | Yes |
| **Risk of bias** | 5 | Specify the methods used to assess risk of bias in the included studies. | Yes |
| **Synthesis of results** | 6 | Specify the methods used to present and synthesize results. | Yes |
| **RESULTS** |  |  |  |
| **Included studies** | 7 | Give the total number of included studies and participants and summarise relevant characteristics of studies. | Yes |
| **Synthesis of results** | 8 | Present results for main outcomes, preferably indicating the number of included studies and participants for each. If meta-analysis was done, report the summary estimate and confidence/credible interval. If comparing groups, indicate the direction of the effect (i.e. which group is favoured). | Yes |
| **DISCUSSION** |  |  |  |
| **Limitations of evidence** | 9 | Provide a brief summary of the limitations of the evidence included in the review (e.g. study risk of bias, inconsistency and imprecision). | No |
| **Interpretation** | 10 | Provide a general interpretation of the results and important implications. | Yes |
| **OTHER** |  |  |  |
| **Funding** | 11 | Specify the primary source of funding for the review. | No |
| **Registration** | 12 | Provide the register name and registration number. | No |

*From:* Page, M. J., McKenzie, J. E., Bossuyt, P. M., Boutron, I., Hoffmann, T. C., Mulrow, C. D., Shamseer, L., Tetzlaff, J. M., Akl, E. A., Brennan, S. E., Chou, R., Glanville, J., Grimshaw, J. M., Hróbjartsson, A., Lalu, M. M., Li, T., Loder, E. W., Mayo-Wilson, E., McDonald, S., … Moher, D. (2021). The PRISMA 2020 statement: An updated guideline for reporting systematic reviews. *BMJ, 372*(71). <https://doi.org/10.1136/bmj.n71>

**Supplemental Material 2: List of Studies Included in the Review**

Becker, F., & Becker, S. (2008a). *Young adult carers in the UK: Experiences, needs and services for carers aged 16–24*. Princess Royal Trust for Carers. http://static.carers.org/files/yac20report-final-241008-3787.pdf

Becker, S., & Becker, F. (2008b). *Services needs and delivery following the inset of caring amongst children and young adults: Evidenced base review*. Commission for Rural Communities. https://www.sussex.ac.uk/about/documents/service-needs-evidence-review-on-young-carers-and-young-adult-carers.pdf

Boumans, N. P. G., & Dorant, E. (2018). A cross-sectional study on experiences of young adult carers compared to young adult noncarers: Parentification, coping and resilience. *Scandinavian Journal of Caring Sciences*, *32*(4), 1409–1417. https://doi.org/10.1111/scs.12586

Canell, A. E., Bashian, H. M., & Caskie, G. I. L. (2020, November). *Do the reasons emerging adults become informal caregivers relate to future willingness to care? A mixed-method study* [Poster presentation]. Annual meeting of the Gerontological Society of America, Online.

Canell, A. E., & Caskie, G. (2019, November). *Emerging adult caregivers: Perceptions of older adult care recipients, quality of contact, and ageism* [Poster presentation]. Annual meeting of the Gerontological Society of America, Austin, TX.

Canell, A. E., & Caskie, G. (2020, August). *Emerging adult caregivers: Quality of contact, ageism, and future caregiving* [Poster presentation]. Annual meeting of the American Psychological Association, Washington, DC.

Cass, B., Brennan, D. D., Thomson, C., Hill, T., Purcal, C., Hamilton, M. G., & Adamson, E. (2011). *Young carers: Social policy impacts of the caring responsibilities of children and young adults*. Social Policy Research Centre. https://www.arts.unsw.edu.au/sites/default/files/documents/1_Young_Carers_Report_Final_2011.pdf

Day, Chantelle. (2015). Young adult carers: A literature review informing the re-conceptualisation of young adult caregiving in Australia. *Journal of Youth Studies*, *18*(7), 855–866. https://doi.org/10.1080/13676261.2014.1001826

Day, Chantelle. (2019). An empirical case study of young adult carers’ engagement and success in higher education. *International Journal of Inclusive Education*. https://doi.org/10.1080/13603116.2019.1624843

Greene, J., Cohen, D., Siskowski, C., & Toyinbo, P. (2017). The relationship between family caregiving and the mental health of emerging young adult caregivers. *Journal of Behavioral Health Sciences & Research, 44*(4), 551–563. https://doi.org/10.1007/s11414-016-9526-7

Grenard, D. L., Valencia, E. J., Brown, J. A., Winer, R. L., & Littman, A. J. (2020). Impact of caregiving during emerging adulthood on frequent mental distress, smoking, and drinking behaviors: United States, 2015–2017. *American Journal of Public Health*, *110*(12), 1853–1860. https://doi.org/10.2105/AJPH.2020.305894

Hamilton, M. G., & Adamson, E. (2013). Bounded agency in young carers’ lifecourse-stage domains and transitions. *Journal of Youth Studies*, *16*(1), 101–117. https://doi.org/10.1080/13676261.2012.710743

Haugland, B. S. M., Hysing, M., & Sivertsen, B. (2020). The burden of care: A national survey on the prevalence, demographic characteristics and health problems among young adult carers attending higher education in Norway. *Frontiers in Psychology*, *10*, article 2859. https://doi.org/10.3389/fpsyg.2019.02859

Jones, L. (2018). *Exploring the resilience and identity of young carers in higher education: A thematic analysis* [Master’s thesis, Manchester Metropolitan University]. Manchester Metropolitan University's Research Repository. https://e-space.mmu.ac.uk/621710/1/Laura%20Jones.pdf

Joseph, S., Sempik, J., Leu, A., & Becker, S. (2020). Young carers research, practice and policy: An overview and critical perspective on possible future directions. *Adolescent Research Review*, *5*, 77–89. https://doi.org/10.1007/s40894-019-00119-9

Kent, E. E. (2020). Time to recognize and support emerging adult caregivers in public health. *American Journal of Public Health*, *110*(12), 1720–1721. https://doi.org/10.2105/AJPH.2020.305951

Kettell, L. (2018). Young adult carers in higher education: The motivations, barriers and challenges involved – a UK study. *Journal of Further and Higher Education*, *44*(1), 100–112. https://doi.org/10.1080/0309877X.2018.1515427

Leu, A., Frech, M., & Jung, C. (2018). Young carers and young adult carers in Switzerland: Caring roles, ways into care and the meaning of communication. *Health and Social Care*, *26*, 925–934. https://doi.org/10.1111/hsc.12622

Levine, C., Hunt, G. G., Halper, D., Hart, A. Y., Lautz, J., & Gould, D. A. (2005). Young adult caregivers: A first look at an unstudied population. *American Journal of Public Health*, *95*(11), 2071–2075. https://doi.org/10.2105/AJPH.2005.067702

Lewis, F. M. (2017). *Who am I?: An exploration of identity development of young adult carers in the United Kingdom and United States* [Doctoral thesis, University of Birmingham]. UBIRA e-theses. https://etheses.bham.ac.uk/id/eprint/8899/1/Lewis18PhD.pdf

Mancini, J., Simeoni, M.-C., Clément, A., Viens, P., & Auquier, P. (2006). A modest impact of cancer on young adult caregivers’ educational plans? *American Journal of Public Health*, *96*(7), 1150. https://doi.org/10.2105/AJPH.2006.087080

Struckmeyer, K. (2013). *Emerging adulthood caregivers: Examining the unique experiences in this understudied population* [Master’s thesis, Arkansas Tech University]. SHAREOK. https://shareok.org/bitstream/handle/11244/45319/Struckmeyer_okstate_0664M_14173.pdf?sequence=1&isAllowed=y

Thompson, C. M., Frisbie, A., Hudak, N., Okamoto, K. E., & Bell, S. (2017). “Understanding” as support for emerging adults whose parents have chronic health conditions: A life-span communication perspective. *Journal of Family Communication*, *17*(4), 301–318. https://doi.org/10.1080/15267431.2017.1330269

**Supplemental Material 3: Included Study Objectives, Measures, Caregiving Context, Results, and Key Findings**

| Author(s) | Objectives/Research questions | Measures | Caregiving context | Results | Key findings |
| --- | --- | --- | --- | --- | --- |
| Becker and Becker (2008a) | - What factors influence the extent and nature of caring? - How do educational experiences affect educational outcomes and employment prospects? - What are the experiences of YAC at college or university? - How are the aspirations of YAC affected by their caring responsibilities? - Do YAC receive a carer’s assessment? - What are the information needs of YAC? - Are there gender differences in the experiences of male and female YAC? - How do young carers projects prepare YC for their service ending and link them into adult carer services? - What supports and services do YAC receive? - What service provisions do YAC need and want? | - Individual semi-structured interview about family background and structure, the needs and services received by the care receiver, the experience of caring, the impacts of caring and protective factors, carers’ support, and their aspirations for the future - Survey | - Care receiver: mother (62.07%), siblings (29.69%), father (6.90%), grandparent (3.45%), uncle (3.45%), partner (3.45%) - Responsibilities: emotional care (under two-thirds), personal and intimate care (under one-third; help the care receiver to wash or take a bath), household task (two-thirds; cooking, cleaning, and shopping), practical support (one-third; paying bills, filling in forms, and writing letters or emails), others (administering or reminding the care receiver to take medication, preparing family meals, collecting library books or special foods, caring for siblings including taking them to and from school, taking the care receiver out, giving medication) - Hours per week: more than 20 hrs (one-quarter), more than 50 hrs (12%) - Caring for: at least two people (20%) | - 5.3% of youth aged 18–24 in the UK are YAC - YAC are people aged 18–24 who provide or intend to provide care, assistance, or support to another family member on an unpaid basis. The person receiving care is often a parent but can be a sibling, grandparent, partner, own child, or other relative who is disabled, has some chronic illness, mental health problem, or other condition (including substance misuse) connected with a need for care, support, or supervision - Providing emotional care was a task that many YAC found particularity arduous and which restricted their participation in other life events, activities, and socializing - Where a parent’s condition deteriorated, the amount of caring increased unless adjustments were made to care arrangements or support materialized from other family members - For YAC looking after siblings, the amount of caring could decrease as their brother or sister matured and they were able to do more for themselves - Just over a third of the sample reported having “strained relationships” with the care receiver - YC are missing school, and they also miss out on career events and advice provided in schools, which reinforces their disadvantage - Some YAC were unable to think long-term about their future because of their existing caring responsibilities - Many YAC were unaware of what help may be available to support their relatives, how to access this and who supplies it - YAC described having an increasing number of other demands on their time alongside their caring responsibilities, be it to do with education, jobs or personal relationships - Understanding and appropriate support from school staff had made a significant difference in terms of engagement and achievement for YAC with caring responsibilities when they were at school - Generally, YAC reported that their experience of college was more positive than school because of its flexibility, its adult-oriented focus, and staff who were more understanding and supportive of young adults with caring roles. However, some YAC had left college prematurely without completing their intended qualifications - YAC at university are required to balance caregiving with their academic studies and learning. Some do this by “caring at a distance” and returning home at weekends or holidays to provide care. Other balance care and study by continuing to live at home and traveling to their local university, enabling them to maintain their regular caring roles - YAC who are studying at university but return home each day to care have substantial and significant caring roles which appear to be no less time-consuming than for those caring and living at home who are not in higher education - YAC who choose to go to their local university and remain as carers either have no one else to undertake their caring roles or don’t wish to disrupt close family ties, and so they return to these responsibilities after their day at university - Of the YAC at university, none were aware of any specific carers support available for them at their university. Similarly, they were often unaware of local services for carers that might be able to support them because traditionally those services have not reached out to university populations - YAC were sometimes late to or absent from school or failed to complete homework on time - Parental encouragement and positive attitudes toward education were important factors that also influenced YAC school attendance and achievement - YAC who participated in a broader range of leisure and community activities were most likely to be university students - YAC often chose friends and/or partners who were sympathetic and understanding toward their situation. Several respondents spoke of the “burden of their maturity” and how this affected their ability to make friends because it had made them different from their peers and restricted their ability to be spontaneous and carefree - Half of our sample said that they felt they had insufficient time for themselves. Life was constantly busy with little free time, time to be alone or opportunities for rest and relaxation and this was particularly the case for those carers who were providing high levels of emotional support or where they were caring for more than one person. This also affected their ability to look for or take on paid work - Most YAC experience significant financial hardships as a consequence of caring and living in a low-income family where there is physical or mental ill health, disability, and alcohol or drug misuse. Family income is very tight, and there is strong evidence of poverty and social exclusion for all family members and of YAC using their own money to subsidies the needs of parents - Just under a third of the sample had recently or were currently engaged in part-time employment as a means to getting additional money for themselves or their family. Others found it too difficult to combine paid work with the conflicting demands of caring - Some unemployed YAC felt very much alone and unsure of how to make progress in accessing the labor market. Low self-confidence and not having the necessary qualifications impeded their success or they simply felt that combining caring and work was not feasible. Lack of transport was also mentioned as a factor, particularly in rural locations, so that they could not search further away for work - Unlike other young people, whose future aspirations are often mediated by money and qualifications, YAC futures are also mediated by their caring responsibilities, particularly where they are caring for a parent, rather than siblings, and where there is no other extended family support available - Emotional pressures and demands for care make it especially hard for YAC to leave home. Others choose not to leave in order to protect siblings from assuming a caring role - Some YAC in the sample also started caring from a very early age, while at primary school (aged 5 or below), and continued caring throughout their childhood, whilst others started caring much later on, at age 17. The majority of the sample started caring whilst they were at primary school, with nine of them being aged 6 or under. In contrast six of the sample started to care, when they were aged 15 or above, during which time they would have been experiencing many of the challenges of transition between adolescence to adulthood - None of the YAC interviewed had regarded themselves as a carer until this was defined for them by someone else, most often a health or social care professional, teacher or young carers service - Factors why YAC were performing caring tasks: the family structure, their position in the family, the quality of the relationship with the person being supported, and the extent to which that person was receiving or accepting services or support from others, either formally or informally - In over half of the cases (n = 15), the head of the household was a lone parent, and so there was either no one else to care or the majority of the care had fallen onto the YAC as the eldest daughter or son. In about a third of cases (n = 7), there were two parents in the household, and in these cases the amount of caring being undertaken by the YAC was generally lower in terms of hours spent caring, with one exception - YAC identified unmet needs of the care receiver, help for which would have reduced the amount of caring they were required to do - YAC who have gone away to live at university clearly have temporary relief from most of their caring responsibilities, but many still provide emotional support, often conducted over the telephone, at a distance, and many say they still worry a lot about the person they support - YAC who lived away at university were doing less physical caring than before - The majority (n = 17) of YAC in the sample were living at home (“co-resident”) with the care receiver - For some YAC, living away from home has enabled them to gain physical and emotional distance from their family for the first time - Relationships at home can become very strained at the point where the young adult is trying to assert greater independence and where caring responsibilities are negatively affecting their studies or employment - In a number of cases, YAC simply had no choice about leaving home either because of their financial circumstances (for example, if they were still at college, unemployed or earning a low wage) and/or because of their caring responsibilities alongside pressure that the person being supported brought to bear - There was some evidence that YAC were expected to take on greater responsibility for caring because they were seen as more mature and able to cope with it - Positive outcomes of caring: the fact YAC could help to make a loved one feel better or cope with daily living; close attachment that YAC had with the care receiver; acceleration of YAC maturity and chance to develop skills for coping in a crisis or complex situations; development of useful life skills through caring like home care, cooking, and dealing with officialdom; development of empathy, caring, sensitivity, and other positive attributes like being more accepting of difference and less judgmental - Caring had directly affected YAC qualifications or career choices consciously or unconsciously - Negative health outcomes of caring: negative emotional well-being, physical ill health - YAC often neglected their own health, and some were overweight due to poor diet and a lack of time to exercise - YAC engage in risk-taking behavior such as unsafe sex, drinking alcohol to excess, and taking drugs - The pressures of caring combined with strained family relationships and isolation and/or bullying were thought to make YAC more prone to misuse alcohol or drugs as a form of “escapism” - YAC ill health can make caring more difficult, tiring, and stressful for YAC - Unless specific recognition and consideration are given to YAC, they are very likely to be ignored by, or remain invisible to, adult service providers in health, social care, and carer support - Not all YAC in the sample had received a young carers service, despite some of them caring for many years on a regular and substantial basis during their childhood and having significant, inappropriate caring roles that had affected their schooling and emotional well-being - Many young carers services are now aware of the “gaps” in service provision for YAC and questioning to what extent they have a responsibility for filling that gap. A few of the sample were accessing “18 plus” or other transitions-related services that were new developments arising from the work of young carers projects. A number of models of provision are emerging which will require evaluation in order to test their effectiveness - All of the YAC in the sample could, in theory, have been accessing support from adult carers services provided by the voluntary or statutory sectors. In fact, very few had ever used an adult carers service and not to any great degree. Carers centers working with (older) adult carers identified a range of factors (or barriers) that made it either unfeasible or more challenging to engage with young adult carers aged 18–24 - When YAC reached 18 years of age, they found themselves unsupported - Action for YC Plus is a new service for carers aged 18–25 years; the York Carers Centre, the Youth Action Wiltshire, the Islington Young Adult Carers Group, and the Hub Young Carers are for carers aged 16–25 years - YAC workers were aiming to improve YAC mental and physical well-being; enhance YAC confidence and self-esteem; promote age-appropriate respite activities; assist YAC to access employment, training, or education; ensure income/benefit maximization; understand and promote choices; access training, and sometimes certification, on aspects of caring and life skills; access a carer’s assessment and additional support services where appropriate | - YAC are involved in a wide range of caring tasks and responsibilities - YAC health needs should not be overlooked - YAC have needs that are related specifically to their caring roles and responsibilities and their experiences and identity as carers - YAC have a need for information, advice, and guidance (e.g., grants, healthy diet, well-being, caring); services and support (e.g., counseling, breaks from caring, contingency planning, public transport, housing); education and training (e.g., guidance, opportunities); activities and peer support (e.g., leisure activities, social networking, social inclusion); and job-seeking support and flexible employment (e.g., career guidance, labor market participation) - There is no specific service for YAC - Eight recommendations for support services development: (1) clearly identify the outcomes of any intervention regarding support services objectives and resources as well as YAC needs; (2) include YAC in the discussion and the planning of intervention during the development process; (3) involve local workers from YC, adult social care and adult carer services in the intervention development; (4) develop partnerships between YC projects and care centers in order to share learning, materials and ideas; (5) involve institutions and organizations (e.g., university, employers) in identifying and engaging with YAC; (6) be more alert to YAC-specific needs and to the way organizations and support services deliver their particular services to them; (7) at the local level, services should provide information about YAC legal rights; and (8) YAC needs and intervention outcomes have to be integrated within the local authority’s carer strategy |
| Becker and Becker (2008b) | Provide an overview, synthesis, and analysis of research and other evidence on YC and YAC in the United Kingdom | *NA* | - Hours per week: more than 20 hrs (one-quarter), more than 50 hrs (12%) | - 3% of youth aged 18–24 in the UK are YAC - There are various factors that push and pull young adults into caring roles, including family illness/disability, attachment and love, co-residency or close proximity, family structure, gender, age, expectations, and poverty, and few, if any, alternatives - Young adults perform a range of caring tasks and take on significant, substantial, and regular responsibilities for other family members. They find it hard to balance other demands made upon them (education, work, friends, relationships, etc.) with their ongoing caring commitments - As YAC get older, there are growing expectations within some families that they should provide care as they are now “adults,” and the care they give is considered less inappropriate as they get older - Many young carers start caring at a very early age and can continue in caring roles for many years – often throughout their childhood (and into adulthood) - YC and YAC are most often co-resident as they live in the same household as the care receiver, particularly if it’s a parent - Some YAC choose not to leave home in order to protect siblings from assuming a caring role - Positive outcomes of caring: development of knowledge; understanding; sense of responsibility; maturity; and a range of life, social, and care-related skills - YAC often feel that they have insufficient time for themselves: life is constantly busy with little free time, time to be alone, or opportunities for rest and relaxation, and this is particularly the case for YAC who are providing high levels of emotional care or caring for more than one person - In most instances, the needs of YC and YAC are relatively modest and do not require very intensive (or very costly) interventions and services. However, each carer will have their own specific needs depending on family circumstances, the nature of the illness/disability and the need for care, family finances, who else can help provide care from within and outside the family, and so on - Many of the young carers projects do not work with carers over the age of 18. This cutoff can cause problems for many young carers who have been in contact with projects for some years, and who, on reaching 18, find themselves without a service or support. In theory, young adult carers could access support from adult carers services provided by the voluntary or statutory sectors. In fact, very few use an adult carers service and then not to any great degree. Adult carers services and centers are generally used by and promoted to much older carers – from around age 40 and over, rather than young adults aged 18–24 - There is a lack of recognition of the needs of young carers once they reach the age of 18, and of YAC who may start caring between the ages of 18–24, and there are very few services or supports available to them during this “transition” between childhood and adulthood and between children’s and adult services - YAC university students were often unaware of local services for carers that might be able to support them because traditionally those carers services have not reached out to university populations - Rural YC and YAC face particular barriers in accessing and receiving services and support, compounded by distance, lack of adequate public transport, isolation, stigma, and lack of privacy. There is a need for authorities and organizations to plan services and support that recognize (a) the impact of rurality and (b) that it will cost more to develop and provide equality of services in rural areas – the “rural premium” - There are ongoing problems with the identification of YC and YAC in schools, colleges, higher education, workplaces, healthcare, and social care settings - Many YAC are unsupported | - Young adults who are most heavily involved in caring are those most likely to need services, support, and assistance to help promote their own health, well-being, education, development, labor market participation, and social inclusion - Young adults report a wide range of negative outcomes associated with caring, including educational problems, difficulties in finding and keeping paid work, and personal/emotional problems, to name a few. Some report positive outcomes, particularly how caring has helped them to foster even closer bonds with parents and other family members, even though these relationships can be difficult and strained at times - There is still a major problem (on the part of professionals working in education, health, and social care) in identifying and assessing young adult carers, and in meeting their legal rights (to assessments, services, and support) |
| Boumans and Dorant (2018) | Explore YAC perceptions of parentification, resilience, and coping compared to non-YAC | - Demographic and family characteristics: gender, age, educational level (university vs. vocational education), family structure (single- or two-parent family), number of children in the family, and birth rank of the respondent (oldest child, middle child, youngest child, only child) - Have had any experience with giving informal care to a family member - Maastricht Parentification Scale (emotional care of parents, buffer between parents, household care of family, financial care of family, instrumental care of siblings, emotional care of siblings) - Brief Resilience scale - Utrecht Coping List (problem-focused, emotion-focused) - Characteristics of the informal care situation (care receiver, health condition of the care receiver, type of caring task performed, presence of caregiving support by another family member or professional, mean number of hours of caregiving per week, and total number of months providing care) | - Care receiver: mother (38.1%), father (23.8%), sibling (7.2%), grandparent (31%), more than one person (19.2%) - Illness/disability: chronic disease (21.6%), physical disability (9.8%), psychological problems (3.9%), intellectual disability (2%), other or combination (62.7%) - Responsibilities: emotional support (75%), domestic help (67.3%), assist with visits (44.2%), personal care (34.6%), nursing care (32.7%), babysit (32.7%), organizing services (23.1%), administrative tasks (15.4%) - Weekly hours: *M* = 12.5, *SD* = 11.06 - Length of caring: *M* = 31.3, *SD* = 36.14 - Status: only caregiver (13.5%), shared with other family members (50%), shared with professionals (7.7%), shared with other family members and professionals (23.1%), otherwise (5.8%) | - YAC were more in vocational education (nurse; *p* < .001) and from a single-parent family than non-YAC (*p* < .001) - No difference between YAC and non-YAC for gender, number of children in the family, and sibling rank - YAC presented among parentification dimensions more emotional care of parents (*p* < .01), household care of family (*p* < .001), and instrumental care of siblings (*p* < .01) than non-YAC - No differences among parentification dimensions between YAC and non-YAC for buffer between parents, financial care of family, and emotional care of siblings - No differences between YAC and non-YAC for the level of resilience and problem-focused coping - YAC had a higher score of emotion-focused coping than non-YAC (*p* < .05) | - YAC felt more engaged in emotional care of their parents and in instrumental parental roles to their siblings than non-YAC - YAC experienced more excessive household care than non-YAC - YAC showed more emotion-focused strategies than non-YAC |
| Canell and Caskie (2019) | - Is percentage of positive or percentage of negative adjectives describing the care receiver correlated with YAC attitudes towards older adults as a group? - Are descriptions of older adult care receivers correlated with quality of contact in a caregiving dyad? | - Fraboni Revised Ageism Scale - Quality of contact (“How would you describe the overall contact with the older adult you are caring for?”) - Adjective list (“Please list five adjectives that describe the older adult you provide care for”) - Demographic: age, gender, education, income, ethnicity, US state, relationship to care receiver, length of caregiving, extent to which caregiving tasks are shared - Demographic of the care receiver: reason for care, dependency, age, and gender | - Care receiver: parents (16%), grandparents (63%), aunt/uncle (5%), friends (3%), neighbor (5%), and others (8%) - Illness/disability: general physical decline (61%), arthritis (37.6%), dementia/Alzheimer's disease (34.3%), hypertension (26.8%), depression/other mental illness (22.1%) - Responsibilities: activity of daily living and instrumental activity of daily living - Status: primary (58.7%), secondary (26.8%), and equal (14.6%) caregiver | - Quality of contact with the care receiver was significantly correlated (*p* < .001) with the percentage of positive and negative adjectives - Scores on the Fraboni Scale of Ageism were significantly correlated (*p* < .01) with the percentage of positive and negative adjectives | - YAC have generally positive perceptions of their older adult care receiver regarding the quality of contact in the caregiving relationship |
| Canell and Caskie (2020) | Examine whether the quality of contact between an YAC and an older adult care receiver moderated the relationship between ageist attitudes and willingness to be a caregiver in the future | - Fraboni Scale of Ageism - Ambivalent ageism scale - Quality of contact (“How would you describe the overall contact with the older adult you are caring for?”) - Contact with older adults scale - Willingness to care (emotional care, instrumental care, and nursing care subscales) | - Care receiver: grandparents (60%) - Care receiver age: *M* = 75.23, *SD* = 8.28 - Illness/disability: dementia/Alzheimer’s disease (35%) - Responsibilities: activity of daily living or instrumental activity of daily living - Status: primary caregivers (60%) | - The interaction term for ageist attitude and quality of contact was significant (*p* < .05) for both instrumental and nursing care - The interaction term for ambivalent ageist attitude and quality of contact was significant (*p* < .01) for both instrumental and nursing care | - As quality of contact decreased, more overt ageist attitudes became more strongly related to lower willingness to provide both instrumental and nursing care - As quality of contact decreased, more ambivalent ageist attitudes were more strongly related to higher willingness to provide instrumental and nursing care |
| Canell et al. (2020) | *NA* | - “Please describe the circumstances that led you to provide unpaid care to this older adult” - Willingness to care (emotional care, instrumental care, and nursing care subscales) | - Care receiver: parents (16%), grandparents (63%), aunt/uncle (5%), friends (3%), neighbor (5%), and others (8%) - Illness/disability: general physical decline, arthritis, dementia/Alzheimer's disease, hypertension, depression/other mental illness - Responsibilities: activity of daily living or instrumental activity of daily living - Status: primary (58.7%), secondary (26.8%), and equal (14.6%) caregiver | - 11 circumstances emerged: care receiver illness (35.5%), family relationship (35.5%), care receiver became dependent (23.8%), proximity (13.7%), only caregiving option (10.1%), reciprocal care (8.9%), availability (8.5%), age-related decline (6.9%), monetary restrictions (6.9%), care receiver desire (6%), community service (4%) - YAC who identified family relationships were less likely to endorse willingness to provide nursing care in the future compared to those who did not identify family relationships (*p* < .05) - YAC who identified care receiver dependency were more likely to endorse willingness to provide instrumental (*p* < .05) and emotional (*p* < .05) care in the future than those who did not identify care receiver dependency | - Care receiver illness and family relationships were the two most common reasons for why emerging adults became informal caregivers - A diversity of experiences led to becoming a carer earlier in life, and may impact future caregiving behaviors |
| Cass et al. (2011) | - Explore experiences and meanings of the caregiving/care-receiving relationship and the policy frameworks which affect those relationships - Explore the diversity of YAC responsibilities and relationships within their families and in their wider kin, friendships and community networks, and their participation in schooling, further education, training, and employment | - Interviews - Questionnaire - Activity sessions | - Care receiver: parent (48%), sibling (48%), other family members (6%) - Illness/disability: physical (70%), mental health (52%), intellectual (39%), long-term illness (36%), sensory (33%), limited mobility (18%), alcohol or drug problem (18%), other (15%) | - Majority of respondents looking after their parents or siblings did not consider their caring role a phase in their life, but rather thought of it as a continuing responsibility - Some young people became YAC because of the deteriorating condition of a relative, or as a result of getting older and being able to take on a greater caring role - YAC expressed a clearer understanding of their role and how it affected their identity and growth, especially their participation in education and employment and future life opportunities - YAC participants talked about growing up as a carer and how they took on more of an emotional caring role as they got older and often had to deal with the changing intensity of their care receiver’s condition and disability - The types of tasks and the intensity of caring were dependent on age, type or nature of care receiver’s conditions, and the structure and dynamics of the family - YAC were more likely to take on additional tasks, including taking the person they cared for to medical appointments, talking with health professionals and other service providers, helping out with household bills and paperwork, and providing emotional support and advice - YC and YAC talked about having very close relationships with the parent and/or sibling for whom they cared, and in many cases, they also said they were close to their whole family - YAC were more likely to comment on their relationship with their parent being “different” and offered reflections on why it was different and how their relationship changed over time - The positive relationships YC and YAC talked about were often presented as the “best things” about being a carer - Many YAC talked about their maturity and sense of self, which they felt was due to their caring role - Another positive aspect which both YC and YAC expressed was a heightened sense of perspective, respect, and acceptance of others - A commonly cited negative aspect of caring was the lack of time to participate in social and recreational activities, as caring was YC and YAC priority - The main coping strategies identified were establishing a routine or schedule, separating their home and school lives, being able to find humor in difficult situations, accepting things as they are, finding the positive aspects (such as love and reciprocity), and making sure they have personal time and space - YAC expressed their frustration with the difficulties of balancing their education and caring task - A small number of YAC expressed strong views about the need to improve awareness about, and support for, young carers in the education system in order to “count them in” - While some YAC found university more difficult to manage because of the increased workload, others found it easier to balance because the system was more flexible and teachers were more understanding than in their high school experience - YAC felt it might be to their advantage for teachers or university tutors and instructors to know about their caring role - YAC, whether they were or were not employed, talked about the difficulty in combining work and care. Most of their concerns were linked to the need for work to be flexible and adaptable, and the need for a “good employer” - Consequences of caring on YAC social lives are greater than for YC - Health consequences of caring were much more profound among the YAC; YAC commented on feeling tired, exhausted, and run down, and some YAC expressed that these impacts had intensified over time - Many YAC discussed the negative effects on their mental health and identified depression, anxiety, stress, and loneliness as conditions they experienced associated with their caring role - YAC experiences and concerns reflect the consequences of intense and prolonged periods of caring - While the majority of YAC either intended to go on to higher education or were already attending university, their caring responsibilities had shaped their choice of institutions (usually the one in closest proximity to home) so they could combine caring and education | - There were similarities and differences between YC and YAC experiences and perspectives - YAC were better able to reflect on their pathway into caring, the changing nature and intensity of caring tasks, and the relationships they had with the person they cared for - Consequences of caring appeared to be more intense at an older age, demonstrated by YAC experiences and perspectives about the negative aspects of caring and the consequences for their future aspirations |
| Chantelle Day (2015) | Provide a foundation for the reconceptualization of YAC as a distinct carer cohort who, without suitable recognition and specifically targeted support, may experience significantly reduced future life opportunities (i.e., career choices, financial and economic security, academic and social engagement, access to formal support services) | *NA* | - Responsibilities: nursing duties (giving medication, changing dressings, and assisting with mobility), personal care tasks (toileting, bathing, and dressing), domestic duties (cleaning, cooking, and laundry), childcare tasks (sibling supervision, lunches, and school run) and emotional support (observing and attending to the care receiver’s emotional and psychological well-being) | - Two-thirds of 388,800 young Australian carers are aged between 18 and 25 years - YAC roles may be endorsed not only due to the presence of illness or disability but also due to notions of non-voluntarism or diminished choice (selection and agentic capacity) | - Australian YAC are recognized neither in policy nor in practice as a distinct carer cohort requiring specifically targeted resources to support their transition towards adulthood |
| Chantelle Day (2019) | Examine YAC patterned actions of engagement and overall outcomes of success in higher education | Quality and quantity of effort YAC invested in: academically challenging activities; within- and beyond-class activities; learning- and teaching-based interactions with staff; broadening educational activities; relationships with peers, teachers, and support services; work-integrated learning experiences | - Care receiver: parents (62%), partner (7%), siblings (31%) - Illness/disability: Huntington’s disease, Parkinson’s disease, Lewy body dementia, paraplegia, bowel cancer, chronic arthritis, depression, bipolar disease, cerebral palsy, muscular dystrophy, autism, intellectual disabilities, various chronic illnesses - Living arrangement: living with the care receiver (92%) | - YAC experienced significant challenges in relation to maintaining regular study routines, keeping up to date with preparatory coursework, and investing quality time and effort into drafting assignments - YAC invested minimal amounts of time on campus, preferring to study at home so they could “keep an eye” on the person to whom they provide care - YAC rarely socialized with peers or staff outside of scheduled class hours and were reluctant to disclose their caring status for fear of being stigmatized as inferior or incompetent students - YAC felt less prepared to enter the workforce and less likely to be successful in securing employment - YAC were less satisfied with their progress, performance, and cumulative grade achievements | - YAC experienced significant challenges at university due to the competing demands of their student and carer roles |
| Greene et al. (2017) | - Examine the relationship of family caregiving responsibilities and the mental health and well-being of young adults - Comparison between non-YAC, past YAC and YAC who both were and are caregivers (current/past YAC) | - Demographic information (age, gender, race/ethnicity, participation in extracurricular activities and clubs, whether they had a part-time job(s), number of hours worked weekly if so) - Center for Epidemiologic Studies of Depression Scale - State-Trait Anxiety Inventory - Rosenberg Self-Esteem Scale - Response to Stress Questionnaire (voluntary disengagement, voluntary engagement, problem-solving) - Caregiving variables (all care receivers for whom they had responsibilities, length of caregiving, all caregiving tasks, time spent providing care) | - Current/past YAC care receiver: parents (47%), grandparents (40.8%) - Current/past YAC responsibilities: activities of daily living (feeding, 39.5%; bathing/showering, 30.3%; dressing, 39.5%; toileting, 26.3%; assistance with walking, 59.2%; changing diapers, 17.1%; none of the above, 22.4%), instrumental activities of daily living (organizing help from others, appointments, 64.5%; translating information, 27.6%; providing emotional support, 82.9%; cleaning/doing laundry, 72.4%; grocery shopping/preparing meals, 72.4%; keeping company, 82.9%; bandaging/assisting with medical equipment, 26.3%; administering medication, 44.7%; other, 36.8%; none of the above, 2.6%) - Current/past YAC hours: 3–5 hrs during a typical school day (48.7%), 3–5 hrs during a typical weekend day (28.9%), more than 8 hrs during a typical weekend day (19.7%) - Current/past YAC length: between ages 16 and 20 (43.5%), between ages 11 and 15 (27.5%) - Current/past YAC cared for: one person (66.7%), two or more persons (33.3%) | - Non-YAC were more likely to engage in extracurricular activities (67.9%) than past YAC (58.0%) and current/past YAC (52.6%) - Current/past YAC were significantly more involved in organizing help from others and coordinating appointments (*p* < .05) as well as administering medication (*p* < .05) than past YAC - Current/past YAC presented more depressive symptomatology (*p* < .01), state anxiety (*p* < .01), and trait anxiety (*p* < .05) than non-YAC - No differences between non-YAC, past YAC, and current/past YAC for self-esteem (*p* = .52) - No differences between non-YAC, past YAC, and current/past YAC for problem-solving and wishful thinking coping strategies (*p* = .05) | - YAC provided care for more hours during a typical weekend day than during a typical school day - YAC most often acted as companions, provided emotional support, and cleaned/did laundry - The burden of caregiving coupled with university pressures and other factors likely makes YAC more vulnerable to psychiatric distress |
| Grenard et al. (2020) | Examine the association between caregiving and mental or behavioral health outcomes among emerging adults | - Personal care (“giving medication, feeding, dressing, or bathing”) and household care (“cleaning, managing money, or preparing meals”) - Weekly hours of care provided - Frequent mental distress (stress, depression, problems with emotions) - Binge drinking, heavy drinking, cigarette smoking, and e-cigarette smoking - Demographic information (age, gender, current insurance, race/ethnicity, highest level of educational attainment, yearly household income, current employment status, state of residence) | - Responsibilities: neither household nor personal, household only, personal only, both household and personal - Weekly hours: less than 8 hrs, 9–19 hrs, 20–39 hrs, more than 40 hrs | - 18.1% of emerging US adults were functioning as caregivers and 13.9% were expecting to become a caregiver within the next 2 years - Male and Hispanic respondents comprised a greater proportion of expectant compared with YAC and non-YAC - YAC were less likely to be students than were expectant and non-YAC - Non-YAC were more likely to report the higher annual household income than expectant and YAC - Frequent mental distress among YAC was 67% higher than expectant (95% CI = 1.28, 2.17) and 50% higher than non-YAC (95% CI = 1.23, 1.82) - Cigarette smoking was more prevalent among YAC than non-YAC (95% CI = 1.21, 1.71), but did not differ compared with expectant - No differences between YAC, expectant, and non-YAC for drinking behaviors and e-cigarette use - Compared with expectant, YAC who reported managing solely personal tasks (95% CI = 1.20, 3.24) or both personal and household tasks (95% CI = 1.43, 2.65) had a significantly higher prevalence of frequent mental distress; the association was not significant for YAC providing household tasks only - Frequent mental distress was more prevalent among YAC providing 8 or fewer hrs, 9–19 hrs, and 40 or more hrs than expectant and non-YAC, but not among those providing 20–39 hrs | - Caregiving among emerging adults is associated with higher frequent mental distress - The association between caregiving and frequent mental distress differed by type of care provided, with personal care showing a stronger association - Even caregiving for minimal time poses a risk of frequent mental distress - There was no evidence that caregiving was associated with problem or risky drinking or e-cigarette use |
| Hamilton and Adamson (2013) | Explore in detail the diversity of YAC responsibilities and relationships; their participation in schooling, further education, training, and employment; and their social relationships, health, well-being, and services needs | - Bounded agency - Socioeconomic and demographic information (family composition and history; circumstances and needs of the person being cared for; the health and disability status and well-being of all family members) - Satisfaction with education, employment, health, and their future - Reported use of services | - Care receiver: mother (63.63%), father (9.09%), siblings and other family members (grandfather and child; 27.27%) - Illness/disability: physical (30.77%), mental (15.38%), intellectual (11.54%), long-term illness (11.54%), sensory (11.54%), limited mobility and others (19.23%) - Length: less than 5 years (16.67%), 5–9 years (50%), 10 years or more (33.33%) | - YAC who had been caring for a shorter duration had similar experiences to other YAC - YAC expressed concern about a lack of understanding, support, or flexibility in the schooling system - YAC in university or vocational education faced challenges in managing caring and study because of increased workload, the logistics of transport and course timetables, and (for some) balancing part-time work - YAC ambition to go to university was not affected by caring even if caring responsibilities influenced YAC choice of institution (proximity of the university to their home), the course they studied, and the hours they were able to attend - YAC asserted that caring itself was a choice they made and they continued to do it because they wanted to do so - YAC, regardless of their employment status, talked about the difficulty of combining work and care - YAC concerns were linked to the need for work to be flexible and adaptable and the importance of having a “good employer” - For some YAC, employment choices were constrained by what they perceived was possible or practical within the demands of their caring responsibilities - Moving away from the family home was made difficult by caring responsibilities and concerns about leaving the care receiver alone or leaving other family members with greater responsibilities - Decisions to leave home also depended on the nature of the care receiver’s illness or disability and the potential intensity of care required in the future - YAC described the physical toll of the manual work and recalled getting sick frequently, as well as feeling exhausted and run down and having difficulty sleeping - Many YAC identified effects of caring on their mental health - YAC concerns about their physical health reflected the consequences of intense and prolonged periods of caring - YAC identified clear gaps in service provision | - Caring was YAC priority - Caring even during a short duration had similar impact with respect to experiences of education, employment, and social life - Caring responsibilities have a negative impact on YAC health and well-being - Compared to YC, YAC major themes were employment, living independently, and health |
| Haugland et al. (2020) | Examine prevalence, characteristics and health outcomes among YAC who provide informal care to family members or others with physical or mental illness, substance misuse, or disabilities | - Demographic information (sex, age, relationship status, accommodation status, immigrant status) - The Hopkins Symptoms Checklist (depression and anxiety) - Sleep problems (number of nights per week they experienced difficulties initiating sleep, difficulties maintaining sleep, and early morning awakenings; daytime sleepiness and tiredness; duration of these sleep problems) - Somatic Symptom Scale-8 (somatic/physical health) - Satisfaction with Life Scale | - Illness/disability: physical or mental illness, disabilities, substance misuse | - Prevalence of 5.5% of YAC among Norwegian students - 81.3% of YAC did not live with the care receiver - Caring was associated with being female (*p* < .001), being single (*p* < .001), having divorced parents (*p* < .001), being an immigrant (*p* < .001), and having financial difficulties (*p* < .001) - Both on weekdays and weekends, a significantly larger proportion of females compared to males spent 2 hrs or more on care responsibilities (*p* < .001) - Both females and males spent more hours caring on weekends than on weekdays - Mental health problems were significantly associated with amount of care responsibilities in a dose-response manner - YAC spending 1 hr or less (44.7% for females and 24.4% for males), and 2 hrs or more (56.4% for females and 31.4% for males) per weekday scored above the cutoff for moderate symptoms of anxiety and depression - YAC presented more insomnia than non-YAC - YAC females spending 1 hr or less (46%), and 2 hrs or more (53.7%) per weekday fulfilled the DSM-V criteria for an insomnia disorder; no dose-response relationship observed for males - Somatic symptom burden was significantly associated with amount of care responsibilities in a dose-response manner - YAC spending 1 hr or less (33.5% for females and 15.9% for males) and 2 hrs or more (46.5% for females and 24.5% for males) per weekday were classified as having a high or very high somatic symptom burden; the magnitude of associations was similar among male and female YAC - Life satisfaction was significantly associated with amount of care responsibilities in an inverse dose-response manner - YAC spending 1 hr or less (29.5% for females and 37.6% for males), and 2 hrs or more (22.1% for females and 31.9% for males) per weekday reported a high or very high life satisfaction; the magnitude of associations was similar among male and female YAC | - YAC spent more time during weekends compared to weekdays on care responsibilities - YAC presented more mental health problems, insomnia, and somatic symptoms, as well as lower satisfaction with life, compared to non-YAC - The number of hours spent on caring was associated with the magnitude of mental health problems, insomnia, somatic symptoms, and dissatisfaction with life in a dose-response pattern |
| Jones (2018) | Explore the underlying dynamics surrounding the development of resilience and positive identities in YAC studying at college and university | Semi-structured interview | - Care receiver: mother (20%), father (40%), brother (20%), grandmother (20%) - Illness/disability: disability (40%), stroke (20%), autism (20%), terminal cancer (20%) - Length: 3 years (20%), 4 years (20%), 8 years (20%), 9 years (20%), 10 years (20%) | - YAC expressed psychological discomfort, as they perceived themselves to be different from their peers - Caring behaviors can influence the development of increased emotional maturity in young adulthood, characterized by some degree of control over feeling states - YAC participate in voluntary work that aims to educate the community about the challenges faced by carers - Caring roles led to the development of specific skills which empowered YAC feelings of competence | YAC belief that they are competent and confidence in looking after themselves and others as a carer is believed to have a positive effect in strengthening their ability to be resilient, flexible, and strong |
| Joseph et al. (2020) | - Provide a critical discussion of the issues and challenges facing YC and YAC researchers in the coming years - Provide directions for how the field now moves forward | *NA* | *NA* | - Some countries have well-developed services and recognition in law, while others are only just beginning to recognize the problem - Without a universal definition, it is difficult to assess prevalence of YAC consistently across studies - Caring can be conceptualized as three concentric circles: “caring about,” which referred to YAC who help in a minimal way (e.g., household chores), not more than many non-carers; “caring for,” which referred to YAC who take on a level of responsibility (e.g., household chores, nursing duties) but not to the point of interfering with their social and educational lives; and “need care,” which referred to YAC who have taken on a high level of responsibility (e.g., household chores, nursing duties, intimate care, emotional care), beyond that of non-carers, and who are enabled to engage in social and education lives | - Provide support for families such that young people do not have to take on caring roles - The term “YAC” is a broad descriptor only, as this is not one single population |
| Kent (2020) | *NA* | *NA* | *NA* | - Lack of support services infrastructure devoted to YAC in the United States - Support services should be coherent with the emerging adulthood period; encourage health-promoting behaviors (e.g., exercises, socialization) and discourage adverse health behaviors (e.g., drinking, smoking) to regulate stress responses; boost functional coping strategies, such as problem-focused, to simultaneously enhance healthier behaviors; and involve the whole family | - It is time to recognize and support YAC |
| Kettell (2018) | - What motivates YAC to apply for higher education? - What barriers do YAC face when considering higher education? - What challenges do YAC experience when attending higher education? | - How does YAC experience split loyalties? - Negotiating the barriers and challenges to learning - Considering support: past, present, and future | - Care receiver: sister (25%), brother (25%), father (25%), mother (25%) - Illness/disability: mental health condition (25%), autism (25%), bipolar disorder (25%), multiple sclerosis (25%) - Cared for: one person (66.67%), two persons (33.33%) | - YAC often felt their loyalties were divided between wanting to be a good student and wanting to be a good carer - For YAC who do choose to disclose their carer status, having a written record, or carer’s passport, may provide a useful resource during challenging times, or when the impact of caring is particularly difficult to negotiate. This could also be considered for use to obtain priority with car parking - Ensuring staff are aware of what support services are available, and understand how to signpost students effectively, may help alleviate the feeling of being “passed on” experienced by some YAC - Encouraging the establishment of YAC societies; as YAC have reduced opportunities to socialize; having a dedicated society may help reduce isolation and provide a sense of belonging | - YAC academic or vocational aspirations were often tempered with the realities of balancing their ambitions with their caring responsibilities |
| Leu, Frech, et al. (2018) | Explore the personal experiences and perceptions of YC and YAC | - Pathways into care (“In addition to your school/education, you care for a sick family member. Could you please tell me how did this come about?” - Nature and intensity of the provided care (“Could you tell me what you do for [your family member]?”) - Living circumstances (“What does a typical day in your life look like at home and in school/education?”) - Family life (“How would you describe living together in your family?”) - Further care arrangements (“How do you organize the care for [your family member] with the other persons involved [in providing care]?”) - Sociodemographic information: gender, language region, family member they supported, impairment of the family member they cared for, family situation, number of siblings, and family background | - Care receiver: mother (71.43%), father (14.28%), siblings (7.14%), grandparents (7.14%) - Illness/disability: physical (28.57%), mental (64.28%), no diagnosis (7.14%) - Responsibilities: emotional support; personal care; sibling care; domestic and household tasks, dealing with financial issues; and coordinating with professionals from healthcare, social service, and other authorities - Living arrangement: live with their family (85.72%), live with their partners (14.28%) | - When YAC had siblings, they took on responsibility for them - Some YAC have difficulties coping with vocational training and higher education while having a caring role - Three ways to become a carer were highlighted: suddenly (abrupt start into their caring role following a significant change in their family structure), gradually (young people tend to take on greater responsibility within the family as they become older), and normal (“normal” process within their family life) - When YAC talked about their situation outside the privacy of home, it was mainly in agreement with the person they cared for or other close family members - When communicating about the illness, several YAC reported that they were often contacted by other family members who wanted to know about the current state of health of the person they looked after - Talking to professionals was often seen as difficult - Getting into contact with healthcare professionals in hospitals seemed to be challenging when YAC were the main carer - Talking to relevant persons in education or vocational training also emerged as an important topic - Talking about an ill family member with friends in vocational training showed positive as well as negative impacts on the young carers’ relationship with their peers - When the family illness was something the YAC did not want to talk about, they avoided social interaction with friends - When having other young carers as friends, YAC valued their exchange and felt supported by their peers | - The tasks YAC are responsible for, depend on the nature of the illness or impairment of the care receiver and the availability of other carers within the family - The intensity of the caring role varies from part‐time to full‐time responsibility - YAC often had difficulties in concentrating and difficulties in focusing on their social life due to worrying about their ill or disabled family member |
| Levine et al. (2005) | Examine the prevalence, characteristics, and responsibilities of YAC who are caregivers for ill, elderly, or disabled family members or friends | *NA* | - Care receiver: grandmother (42.2% for Harvard/UHF/VNS and 24.1% for NAC/AARP), mother (7.4% for Harvard/UHF/VNS and 15.4% for NAC/AARP) - Responsibilities: instrumental activities of daily living (98.2% for Harvard/UHF/VNS and 99.8% for NAC/AARP; shopping, housework, transportation, and meal preparation), managing finances (11.7% for Harvard/UHF/VNS and 53.2% for NAC/AARP), arranged services (8.3% for Harvard/UHF/VNS and 16.5% for NAC/AARP), activities of daily living (53.1% for Harvard/UHF/VNS and 50.4% for NAC/AARP; bathing, toileting, feeding, and dressing), helping the care receiver to get off the bed (about 39%), dressing (27%), toileting (23.3%), assistance in taking medication (26.8% for Harvard/UHF/VNS and 37.5% for NAC/AARP) - Hours: 8–20 hrs a week (33% for Harvard/UHF/VNS), 9–20 hrs (25.4% for NAC/AARP), 21 or more hrs (23.6% for Harvard/UHF/VNS and 20.7% for NAC/AARP) - Length: 1–4 years (38.7% and 28%), 5–9 years (19.2% and 14.5%), 10 years and more (8.7% and 5.5%) | - The Harvard/UHF/VNS study found the prevalence of 18%, whereas the NAC/AARP study found 12% - YAC were caring for a female (65.8% for Harvard/UHF/VNS and 59.4% for NAC/AARP) - YAC were, in general, caring for someone two generations older, as they cared more often for a grandmother than a mother - High percentage of YAC males (57.3% for unweighted Harvard/UHF/VNS and 51% for NAC/AARP) - The care receiver had many health problems - YAC are less likely than older carers to do the most intimate kinds of personal care - The most common activities of daily living were getting out of bed, dressing, and toileting - YAC have a variety of coping strategies, as reported including prayer (57.4%), talking to family and friends (54.1%), and using the internet (34.5%) - 16.8% of YAC said that they had difficulty obtaining medical help for the care receiver, whereas 72.1% said that they had difficulty obtaining nonmedical help, defined as home care aides or other assistance; 11.1% reported problems in both areas | - Many YAC are male, which refutes the prevalent gender stereotype but also raises serious questions about whether the types of support services that have traditionally targeted older women are appropriate for them |
| Lewis (2017) | Understand the potential impact that caring responsibilities may have upon the development of identifying for YAC living in the United Kingdom and United States | Semi-structured interview (“Tell me about a typical day in your life”) | - Care receiver: multiple family members (44.44% for UK and 39.28% for USA), parents (62.96% for UK and 25.0% for USA), siblings (11.11% for UK and 28.57% for USA), grandparents (3.7% for UK and 21.42% for USA) - Illness/disability: mental illness (22.22% for UK and 14.28% for USA), substance use issues (11.11% for UK and 3.57% for USA) - Responsibilities: physical, emotional, intimate, household management, and financial support - Length: before age 16 (88.88% for UK and 57.14% for USA), after age 16 (11.11% for UK and 39.28% for USA) | - Before official identification, YAC thought of their caregiving as the fulfillment of their membership in their family and an enactment of their normal family practice - Before official identification, YAC were understood solely through their familial role (i.e., child, grandchild, or sibling) - YAC may be reluctant to openly associate themselves with the YAC label because of courtesy stigma and the threat of mistreatment from others - YAC can maintain their identification with the YAC label while also actively considering the appropriate time, setting, and audience to use the label verbally - YAC engagement in formal support services may indicate their prioritized need for support, rather than their identification with the YAC label - Young people are likely to identify as YAC if they provide care for both a sibling and their parent, or alternatively, if they serve as the family’s sole carer - Responsibility for multiple family members across generations may lead young people to feel as if they possess more responsibility, thus strengthening their identification as a carer - YAC in the United States may believe that an association with the YAC identity may be of little value to their lives - Recognition of YAC family contributions as care helped to validate their experiences of young caring - YAC providing care for siblings were less likely to be engaged in a formal support service | - YAC did not view their contributions to their family as care, but rather as a function of their membership in a family - YAC may partially accept the YAC identity out of concern for negative consequences, like bullying and discrimination in connection to their caring role - The destigmatization of disability, illness, and young caring would likely be a great benefit to reducing the negative reaction YAC may face when associating with a young carer identity - Identification as YAC in the United States will not typically lead to an opportunity to receive supportive services |
| Mancini et al. (2006) | *NA* | *NA* | - Care receiver: mother (100%) - Illness/disability: breast cancer (100%) | - Maintaining the household often caused fatigue - YAC insomnia was related to worry - YAC frequently missed classes because of their caring responsibilities at home or at hospital - Giving up social activities and staying at home to care led YAC to spend more time studying | Modest impact of their caregiving role on their educational life |
| Struckmeyer (2013) | - Explore the characteristics of YAC - Determine to what extent life transitions and trajectories influence psychological affect and burden in caregivers - Determine if YAC merit considerations as a distinct group in the classification of family caregivers | - Caregiving activity - Caregiver Well-Being Scale short form - Zarit Burden Interview - Positive and Negative Affect Schedule - Health literacy - Life experiences survey - Open-ended questions (“Before today, did you identify yourself as a caregiver? Why?”, “Is there anything(s) [extracurricular activities, employment, aspirations, etc.] that you have had to alter or give up since taking your role a caregiver? Please list”) | - Care receiver: siblings (21.8%), parents (9.1%), cousin (1.8%), grandparent (12.7%), great aunt/uncle (3.6%), niece/nephew (7.3%), child (1.8%), stepparent (1.8%, friend (27.3%), girlfriend (3.6%), roommate (1.8%), classmate (1.8%), babysat (5.5%) - Responsibilities: activities of daily living and instrumental activities of daily living - Length: less than 6 months (17.8%), 6 to 11 months (11.9%), 1 to 3 years (12.7%), 3 to 5 years (12.7%), more than 5 years (16.9%), do not know (8.5%), choose not to answer (3.4%) - Status: current caregivers (44.1%), past caregivers (90.7%), and caregiver to multiple care receivers (31.4%) | - Emerging adults who are unemployed (56.8%), single (78.8%), female (78%) are more likely to provide care - YAC who occupy the secondary and tertiary caregiving roles can still experience strain or overload - Caregiving activity was positively correlated with caregiver burden (*p* < .001) - No significant correlation was found between caregiving activity and negative psychological affect - No significant correlation was found between caregiving activity and positive psychological affect | - Caring activity was not correlated with well-being or psychological affect - Even though caregiving was defined at the beginning of the study, YAC either reported that they did not identify themselves as a caregiver due to familial obligations or incorporated their own views and past experiences into the definition of a caregiver |
| Thompson et al. (2017) | Over the course of their parent’s chronic illness, how do YAC perceive they provide(d) support to parents with chronic health conditions? | - YAC experiences supporting a parent with a chronic health condition (“Can you tell me how you were providing support to your parent at this point? What does it look like? What is happening here? Why did you put [percentage] support for this moment in time?”) | - Care receiver: mother (including one grandmother; 55%), father (including one stepfather; 45%) - Illness/disability: cancers (14.57%), mental health conditions (13.54%), blood-related illnesses (9.38%), heart conditions (8.33%), diabetes (8.33%), colon conditions (6.25%), chronic pain (6.25%), others (lupus, cerebral palsy; 41.68%) - Responsibilities: verbal and nonverbal behaviors (emotional, showing concern or giving affection); instrumental or tangible behaviors (giving money or helping around the house); and informational behaviors (advice or information) | - Understanding as something YAC both have and communicate - Understanding as something YAC have as a form of knowledge (knowledge through classroom experiences, from parents, personal experiences) - Understanding as knowledge is a kind of recognition of what the health condition is and how it affects YAC parents - Understanding as something YAC have as a form of acceptance (pushing/challenging parent, role expansion and reversals, witnessing) - Acceptance represents YAC acknowledgment that their parents’ chronic health condition is a permanent fixture in their own, their parents’, and their family’s lives; parents are then seen as imperfect and fallible - Understanding as something YAC do as a form of communication (through reciprocating support, sacrificing, being obedient, avoiding sensitive topics, projecting emotional strength) | - Having and communicating understanding differ from typical activities reported in caregiving research - “Understanding” differs from traditional forms of social support and was an important type of support YAC provided - Understanding involves a level of awareness about and concern for parents not captured by verbal and nonverbal behaviors - Some YAC believed that their parent’s health condition brought them closer to their parent, even when it presented conflict |

Note: *NA* = not available. YC = Young carers.
